# Supplementary material for: Spectral signatures of excess-proton waiting and transfer-path dynamics in aqueous hydrochloric acid solutions
Source: Nat Commun. 2022 Jul 21;13:4210. doi: 10.1038/s41467-022-31700-x (PMC9304333; doi:10.1038/s41467-022-31700-x)
Supplement: Supplementary file 1 — Supplementary Information [file 41467_2022_31700_MOESM1_ESM.pdf]

Supplementary Information
Spectral signatures of excess-proton waiting and transfer-path dynamics in aqueous hydrochloric acid solutions

Florian N. Brünig,[1] Manuel Rammler,[1] Ellen M. Adams,[2] Martina Havenith,[2] and Roland R. Netz[1]

[1]*Freie Universität Berlin, Department of Physics, 14195 Berlin, Germany*

[2]*Ruhr-Universität Bochum, Department of Physical Chemistry II, 44780 Bochum, Germany*

Supplementary Methods

Supplementary Methods 1:

Kramers-Kronig estimate of complex index of refraction

Our THz spectroscopic experiments measure the frequency dependent extinction coefficient, $\alpha_{\text{solution}}(\omega)$, defined by the Beer-Lambert law

$$\alpha_{\text{solution}}(\omega) = \frac{1}{d} \ln \left(\frac{I_{\text{water}}(\omega)}{I_{\text{solution}}(\omega)} \right) + \alpha_{\text{water}}(\omega), \tag{1}$$

where d is the sample thickness, $I_{\text{water}}(\omega)$ and $I_{\text{solution}}(\omega)$ are the experimental transmitted intensities of the water reference and the sample. $\alpha_{\text{water}}(\omega)$ is the extinction coefficient of bulk water and is taken from literature [1]. For comparison to the simulated data the power loss, i.e. the absorption spectrum, proportional to the imaginary part of the dielectric susceptibility $\chi''(\omega)$, has to be computed from the experimental extinction coefficient.

The extinction coefficient $\alpha(\omega)$ is related to $n''(\omega)$, the imaginary part of the index of refraction, via

$$\alpha(\omega) = \frac{2\omega n''(\omega)}{c}, \tag{2}$$

where c is the speed of light in vacuum.

The complex dielectric susceptibility $\chi(\omega)$ is related to the complex index of refraction $n(\omega)$ as [2]

$$n^2(\omega) = [n'(\omega) + i\, n''(\omega)]^2 \tag{3}$$

$$= 1 + \chi(\omega) = 1 + \chi'(\omega) + i\chi''(\omega), \tag{4}$$

from which follows

$$\chi'(\omega) = n'^2(\omega) - n''^2(\omega) - 1 \tag{5}$$

$$\chi''(\omega) = 2n'(\omega)n''(\omega). \tag{6}$$

The missing real part of the index of refraction $n'(\omega)$ is related to the imaginary part $n''(\omega)$ by the Kramers-Kronig relation [3]

$$n'(\Omega) = n'(\infty) + \frac{2}{\pi}\text{p.v.} \int_0^\infty \frac{\omega n''(\omega)}{\omega^2 - \Omega^2} d\omega, \tag{7}$$

where p.v. denotes the Cauchy principal value and $n'(\infty)$ is the real index of refraction at the upper boundary of the integral. In practice, since experimental data is only available in a limited frequency range, extrapolation beyond the data range may improve the Kramers-Kronig inversion and a so-called 'anchor point', obtained from an independent measurement, can be used to replace $n'(\infty)$ [4]. For measurements in the infrared (IR) regime, a good estimate for $n'(\infty)$ is obtained by using the real index of refraction measured in the visible [5].

We perform the Kramers-Kronig transform using a set of Ansatz functions for the index of refraction, each consisting of a real part $l_i'(\omega)$ and an imaginary part $l_i''(\omega)$, which follow approximately from a Lorentz oscillator model for the dielectric susceptibility [5]

$$l_i''(\omega) = \frac{A_i^2 \beta_i \omega}{(\omega_{0,i}^2 - \omega^2)^2 + \beta_i^2 \omega^2} \tag{8}$$

$$l_i'(\omega) = \frac{A_i^2 (\omega_{0,i}^2 - \omega^2)}{(\omega_{0,i}^2 - \omega^2)^2 + \beta_i^2 \omega^2}, \tag{9}$$

and extrapolate beyond the range of the available experimental data. We fit the imaginary index of refraction obtained from the experimental extinction coefficient $\alpha(\omega)$ via eq. (2) as

$$n''(\omega) = \sum_i l_i''(\omega), \tag{10}$$

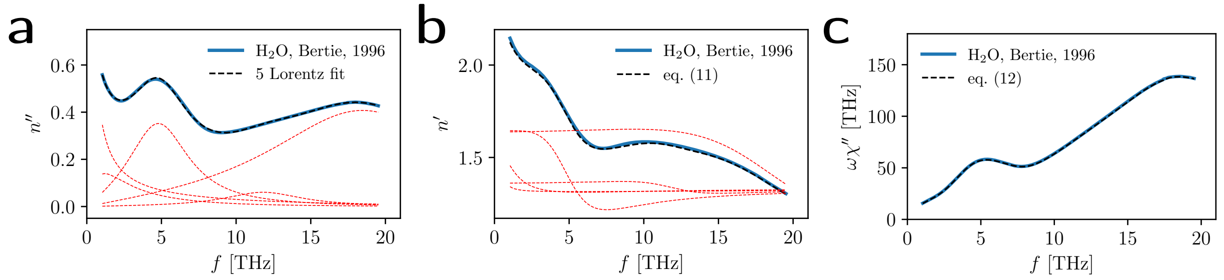

Supplementary Figure 1. Example of the calculation of the absorption spectrum $\omega\chi''$ from refractive index data. The blue solid lines show literature data of water [1]. **a:** Imaginary part of the index of refraction n'' and a fit (black broken line) as a sum of five Lorentz-type fit functions eq. (8) (red broken lines). **b:** Real part of the index of refraction n' and the estimate (black broken line) obtained from the Kramers-Kronig transform eq. (11) of the fit in a. The real parts of the individual fit functions according to eq. (9) are shown as red broken lines, shifted by the offset $n'_{\text{lit.}} = 1.33$. **c:** Absorption spectrum $\omega\chi''$ and the estimate (black broken line) that is computed from the original data for n'' in a and the estimate for n' in b.

where the $l_i''(\omega)$ contain fit parameters A_i, $\omega_{0,i}$ and β_i. The real index of refraction is then given by

$$n'(\omega) = n'_{\mathrm{lit.}} + \sum_i l_i'(\omega), \tag{11}$$

where the constant real index of refraction in the visible $n'_{\mathrm{lit.}} = 1.33$ is taken from the literature, which has been shown to be in general a good approximation for biological matter [5]. The absorption spectrum, $\omega\chi''(\omega)$, which is eventually used to compare to the simulation data, is computed using eqs. (2) and (6) [6] as

$$\omega\chi''(\omega) = c\,\alpha(\omega)n'(\omega), \tag{12}$$

where $\alpha(\omega)$ is the original data and $n'(\omega)$ is parametrized according to eq. (11). As a benchmark example, Supplementary Fig. 1 illustrates the result of this procedure applied to literature data of water [1] in the regime $10\,\mathrm{cm}^{-1}$ to $600\,\mathrm{cm}^{-1}$, for which the experimental THz spectra are obtained. In Supplementary Fig. 2a the fits to the experimental THz data of aqueous hydrochloric acid (HCl) solutions in the range $2\,\mathrm{M}$ to $10\,\mathrm{M}$ (colored solid lines) are shown as black broken lines, that are used to estimate the real part of the refractive index, shown in Supplementary Fig. 2b as broken colored lines. The fit parameters are reported in Supplementary Tab. I. Eventually the resulting absorption spectra, shown in Supplementary Fig. 2c, are used to compare to simulation data in the main text.

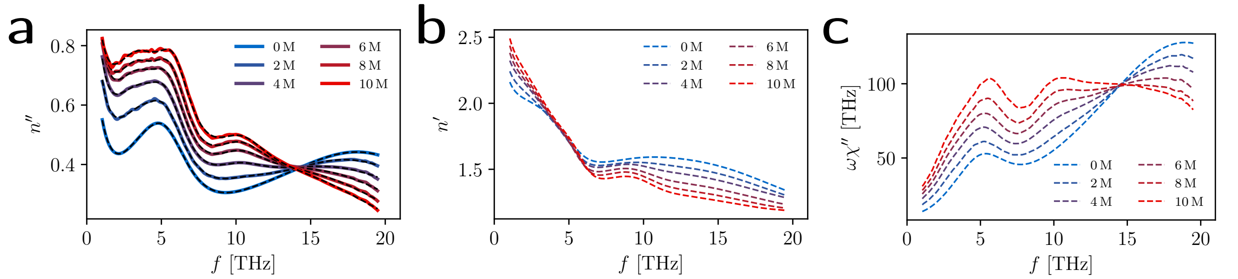

Supplementary Figure 2. Calculation of the absorption spectra $\omega\chi''$ from experimental THz extinction coefficient spectra of aqueous HCl solutions at various concentrations. **a:** Imaginary part of the index of refraction n'' computed directly from the experimental absorption spectra (colored solid lines) and fits (black broken lines). The pure water data (blue solid line, 0 M) is taken from literature [1]. Each fit is a sum of five Lorentz-type fit functions eq. (8) with fit parameters reported in Supplementary Tab. I. **b:** Estimates of the real part of the index of refraction n' (colored broken lines) obtained from the fits in a according to eq. (11). **c:** Absorption spectra $\omega\chi''$ (colored broken lines) that are computed from the original data for n'' in a and the estimates for n' in b.

Supplementary Table I. Fit parameters of the five Lorentz-type fit functions eq. (8), that are used to fit the imaginary part of the index of refraction n'' of aqueous HCl solutions at various concentrations in Supplementary Fig. 2A.

	A_i [THz]	$\omega_{0,i}/(2\pi)$ [THz]	β_i [THz]
0 M			
	0.679	1.749	53.66
	0.3628	4.298	30.04
	0.4378	5.653	26.39
	0.1594	11.81	41.97
	1.206	20.59	120.3
2 M			
	1.008	2.552	141.7
	0.4925	4.314	34.67
	0.3729	5.58	22.37
	0.2535	11.01	40.95
	1.121	20.16	117.5
4 M			
	0.7589	1.301	41.06
	0.5611	4.178	35.08
	0.3975	5.616	21.92
	0.2813	10.49	32.56
	1.175	19.82	137.2

	A_i [THz]	$\omega_{0,i}/(2\pi)$ [THz]	β_i [THz]
6 M			
	0.9225	2.048	81.82
	0.5483	4.082	32.04
	0.4036	5.7	20.58
	0.3602	10.55	33.78
	1.04	18.67	118.9
8 M			
	0.7904	1.703	51.2
	0.5899	4.124	32.88
	0.4266	5.845	20.53
	0.414	10.55	34.43
	0.9807	17.85	112
10 M			
	0.85	2.035	72.92
	0.6844	4.483	39.35
	0.3892	6.035	19.1
	0.3797	10.6	31.25
	0.9387	17.08	109.7

Supplementary Methods 2:
Excess-proton identification

The identification of excess protons from trajectory data is a non-trivial task since the notion of an excess proton itself is subtle. While for the idealized structure of a Zundel cation it is possible to identify one proton that is qualitatively different from the others, in that it is symmetrically shared between two oxygen atoms, such a discrimination is impossible for an ideal symmetric Eigen cation from a configuration at a single time. Certainly it is possible to simply determine the protons that are the least closely associated to any oxygen atom in the simulation at each time step and call them excess protons. This is a perfectly reasonable choice when examining static properties of excess protons, as for example the radial distribution functions (RDFs) in Supplementary Note 4. Marx et al. [7] used a similar definition for the calculation of excess proton probability distributions in ab initio molecular dynamics (MD) simulations of protonated water.

In this work, however, we are interested in dynamical properties of excess protons. Since we aim to track and examine protons during their transfer between water molecules, including when they stay close to an oxygen atom, a selection based on a static geometric criterion would not suffice. Due to fluctuations of protons inside a hydronium molecule, also called 'special pair dance' [8, 9], a criterion based on instantaneous interatomic distances would lead to the disruption of otherwise continuous proton-transfer trajectories. Instead, similar to Calio et al. [9], we record the trajectories of protons that could potentially transfer from one water to a neighboring one and later discard those that did not. In fact, we are selecting *protons that transfer from one water molecule to another* and call them excess protons for simplicity.

Selection of transfer candidates

Ignoring the possibility of spontaneous autoionization of water molecules, proton transfer can only occur from one water molecule to another if the former has an extra proton associated to it, i.e. if it is a hydronium molecule. For the identification of hydronium molecules we use a geometric criterion: Each oxygen atom gets assigned its closest two protons which together form water molecules. The remaining protons are then assigned to the water molecule with

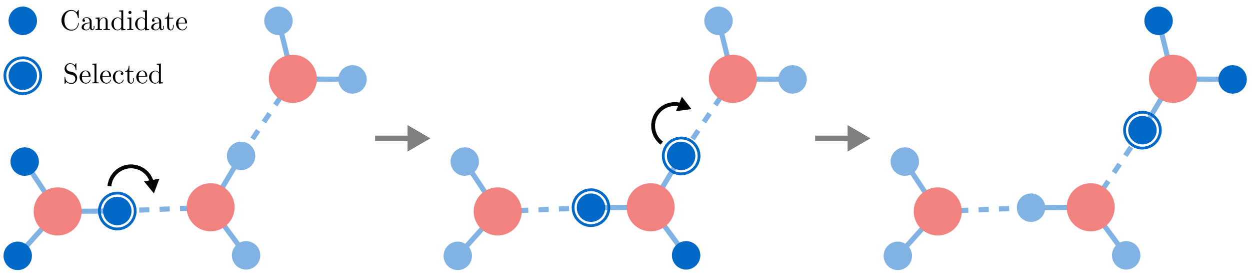

Supplementary Figure 3. Illustration of three consecutive snapshots during a proton transfer process. Transfer candidates and selected protons are highlighted. It becomes apparent why two of the protons belonging to one hydronium molecule are selected at the same time as transfer protons (see second snapshot). Such overlapping trajectories lead to the fact that our conditions select more protons than there are excess protons in the simulation.

the closest oxygen atom and together make up a hydronium molecule. All protons that belong to such a hydronium molecule are considered *candidates* for proton transfer. Furthermore, we exclude those candidates whose distance to their closest chloride ion is smaller than that to their second closest oxygen atom. These protons reside between a chloride ion and an oxygen atom (as opposed to residing between two oxygen atoms as most hydrogen atoms do) and do not exhibit proton transfer between water molecules.

5.5%, 11.1% and 16.4% of all protons fulfill these conditions in the 2 M, 4 M and 6 M simulation, respectively. This means that on average 28.9, 56.4 and 78.4 protons, respectively, are being selected as transfer candidates at each time step, which is a little less than three times the number of excess protons $N_{\mathrm{H}^+} = 10, 20$ and 30 in each simulation. This is to be expected, since each excess proton corresponds to one hydronium molecule which in turn holds three protons that are *part of* H_3O^+. The difference to $3N_{\mathrm{H}^+}$ is due to the excluded protons that are located between an oxygen atom and a chloride ion.

Elimination of trajectories without transfer

The conditions explained above define proton trajectories that potentially contain proton transfers between two water molecules. In order to further reduce the number of protons to those that actually contribute to proton transfer, the trajectories are filtered by another - dynamical - condition that selects only those trajectories which contain a transfer, defined

by a change of the proton's closest oxygen atom (the same criterion was used by Calio et al. [9]). This last condition leaves $\sim 50\%$ of the previously selected protons in each simulation that actually contribute to the analysis, which is still more than $N_{\mathrm{H+}}$ selected protons per time step because some trajectories overlap. This is the case since during the lifetime of a hydronium molecule, two of its protons might be tracked at the same time. One proton which transfers to initially create the respective hydronium molecule and one that transfers away from it later on. Figure 3 illustrates such a situation.

Supplementary Methods 3:

Fits of the free-energy landscape

The two-dimensional (2D) free energy landscape spanned by R_{OO} and d shown in Fig. 4a in the main text, is fitted for cuts through the free energy along d. As in the main text in Fig. 4, here in Supplementary Fig. 4a, cuts along d are shown for $R_{OO} = 2.39\,Å$, where the barrier just vanishes (blue solid line), $R_{OO} = 2.42\,Å$, where the absolute barrier height is minimal (red solid line), and $R_{OO} = 2.51\,Å$, for which the global minima of the 2D free energy are obtained (green solid line). Additionally, some results for other values of R_{OO} are shown as gray solid lines. The data is fitted by the quartic expression $F(d) = F_{d=0}(1 + \gamma_2 d^2 + \gamma_4 d^4)$, shown as black broken lines in Supplementary Fig. 4a with the fit parameters reported in Supplementary Tab. II. In Supplementary Fig. 4b the fit parameters γ_2 (gray solid line) and γ_4 (gray broken line) are plotted over R_{OO}. The vertical colored lines indicated the respective values of R_{OO} shown in Supplementary Fig. 4a. The barrier vanishes for $\gamma_2 > 0$, which defines the blue solid line. At the position where the absolute barrier height is minimal, indicated by the red solid line, the quartic fit parameter γ_4 is maximal.

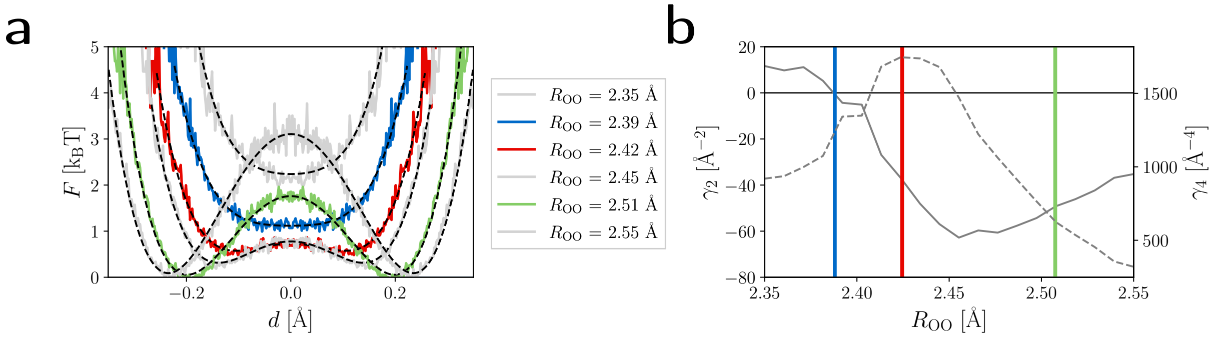

Supplementary Figure 4. Cuts along d through the two-dimensional free energy shown in the main text in Fig. 4a and fitted to $F(d) = F_{d=0}(1 + \gamma_2 d^2 + \gamma_4 d^4)$. (**a**) Cuts at various values of R_{OO} are shown as solid lines and fits as black broken lines with fit parameters reported in Supplementary Tab. II. (**b**) The fit parameters γ_2, shown as a gray solid line, and γ_4, shown as a gray broken line, are given over R_{OO}.

Supplementary Table II. Fit parameters for the fits shown in Supplementary Fig. 4a using the quartic expression $F(d) = F_{d=0}(1 + \gamma_2 d^2 + \gamma_4 d^4)$.

R_{OO} [Å]	$F_{d=0}$ [$k_B T$]	γ_2 [Å^{-2}]	γ_4 [Å^{-4}]
2.35	2.24	11.63	919.7
2.39	1.12	7.78	1099
2.42	0.73	-38.29	1747
2.45	0.78	-62.25	1625
2.51	1.76	-49.17	623.5
2.55	3.11	-35.24	319.6

Supplementary Methods 4: Wiener-Kintchine theorem

The correlation function $C_{xy}(t)$ of two stochastic processes $x(t)$ and $y(t)$ limited to the time interval $[0, L_t]$ is efficiently computed from the Fourier-transformed expressions $\tilde{x}(\omega)$ and $\tilde{y}(\omega)$ according to

$$C_{xy}(t) = \frac{1}{2\pi(L_t - t)} \int_{-\infty}^{\infty} d\omega \; e^{-i\omega t} \tilde{x}(\omega)\tilde{y}^*(\omega), \tag{13}$$

where the asterix denotes the complex conjugate. This is known as the Wiener-Kintchine theorem [10]. Both sides of eq. (13) are Fourier-transformed to give

$$\int_{-\infty}^{\infty} dt \; e^{i\omega t} \; L_t \left(1 - \frac{t}{L_t}\right) C_{xy}(t) = \tilde{x}(\omega)\tilde{y}^*(\omega), \tag{14}$$

which in the limit of large L_t reduces to

$$\widetilde{C}_{xy}(\omega) = L_t^{-1} \tilde{x}(\omega)\tilde{y}^*(\omega). \tag{15}$$

Eq. (13) can be derived starting off with the definition of the correlation function

$$C_{xy}(t) = \frac{1}{L_t - t} \int_0^{L_t - t} dt' \; x(t' + t) y(t'). \tag{16}$$

Defining $x(t), y(t) = 0$ for $t \notin [0, L_t]$, the integral bounds can formally be extended

$$C_{xy}(t) = \frac{1}{L_t - t} \int_{-\infty}^{\infty} dt' \; x(t' + t) y(t'), \tag{17}$$

and making use of the convolution theorem

$$\begin{aligned} C_{xy}(t) &= \frac{1}{4\pi^2(L_t - t)} \int_{-\infty}^{\infty} dt' \int_{-\infty}^{\infty} d\omega \; e^{-i\omega(t+t')} \tilde{x}(\omega) \int_{-\infty}^{\infty} d\omega' \; e^{-i\omega' t'} \tilde{y}(\omega') \\ &= \frac{1}{4\pi^2(L_t - t)} \int_{-\infty}^{\infty} d\omega \; e^{-i\omega t} \tilde{x}(\omega) \int_{-\infty}^{\infty} d\omega' \; \tilde{y}(\omega') \int_{-\infty}^{\infty} dt' e^{-it'(\omega + \omega')} \\ &= \frac{1}{4\pi^2(L_t - t)} \int_{-\infty}^{\infty} d\omega \; e^{-i\omega t} \tilde{x}(\omega) \int_{-\infty}^{\infty} d\omega' \; \tilde{y}(\omega') \; 2\pi\delta(\omega + \omega') \\ &= \frac{1}{2\pi(L_t - t)} \int_{-\infty}^{\infty} d\omega \; e^{-i\omega t} \tilde{x}(\omega) \tilde{y}(-\omega), \end{aligned} \tag{18}$$

noting that $\tilde{y}(-\omega) = \tilde{y}^*(\omega)$ for a real function $y(t)$ in order to obtain eq. (13).

Supplementary Notes

Supplementary Note 1:

Alternative methods for simulation and characterization of excess-proton dynamics

It is known that nuclear quantum effects (NQEs), specifically zero-point effects, significantly influence distributions of excess protons in water [7, 9, 11]. While the techniques for simulating NQEs have significantly advanced in recent years, the accurate calculation of dynamical properties, which is the focus of this study, remains an active field of research [12]. There are some subtle questions how the time-dependent polarization correlation functions of decomposed excess-proton trajectories, which form the basis of our spectroscopic analysis, would be extracted from simulation data encompassing NQEs, in particular, it is unclear whether the common approaches taken by centroid or ring-polymer MD are directly applicable. Most of all, it is noteworthy that previous studies found no significant differences between IR spectra computed from simulations with and without NQEs below $3000\,\mathrm{cm}^{-1}$ [11, 13], which could mean that the neglect of NQEs might have less severe consequences for time-dependent correlation functions than it has for spatial distributions of excess protons. We therefore interpret the good agreement between simulated and experimental spectra in the THz regime in fig. 2c and in the mid-IR regime in fig. 1c as a validation of our chosen simulation techniques. Besides, our neglect of NQEs allows us to generate long trajectories that improve statistics and therefore the quality of our spectra, particularly down in the THz regime.

In Supplementary Note 4 we extract radial distribution functions involving excess protons and chloride ions from our simulation trajectories and obtain good agreement with previous simulations and experimental data [14–18].

In Supplementary Note 7 we show that our transfer-waiting time distributions in fig. 5f correspond quite closely to hydronium-oxygen continuous-identity auto-correlation functions [19], which were previously introduced to characterize proton-transfer time scales. The same correlation functions, but with the fast back-and-forth excess-proton transfer events between the same two water molecules of the transient $H_5O_2{}^+$ complexes removed, have

been used to interpret the long time scales of uni-directional proton transfer, which reflects signatures observed in 2D IR experiments at time scales of 1 ps to 2 ps [19–21]. We note that back-and-forth proton-transfer events that occur within transient ${H_5O_2}^+$ complexes are spectroscopically relevant and therefore must be included in the prediction of spectra. The longer time scale of uni-directional proton transfer at 1 ps to 2 ps contributes in linear absorption spectra at frequencies below 1 THz.

In Supplementary Note 8 we determine the diffusivities of excess protons and water molecules and obtain similar results as previous simulations [13, 16–19, 22]. Compared to the experimental data, the absolute diffusivities of excess protons D_{H^+} and water oxygens D_O are smaller by a factor of about three, but their ratios, given by $D_{H^+}/D_O = (3.0 \pm 0.8), (4.3 \pm 1.3), (3.8 \pm 0.8)$ for 6 M, 4 M, 2 M HCl, respectively, are albeit large errors in satisfactory agreement with the experimental ratios of about $D_{H^+}/D_O = 1.5, 2.3, 3.0$ for similar concentrations [23].

It has been shown that proton-transfer events are caused by subtle structural changes in the excess-proton solvation environment, for example the hydrogen-bond structure in the second solvation shell [11, 13, 18, 22]. Based on our simulations, we confirm that the suggested hydrogen-bond asymmetry coordinate can indeed be used to predict the excess proton that is most likely to transfer to a neighboring water among the three transfer candidates within a hydronium ion [11]. Along these lines, the presence of a fourth water molecule that forms a hydrogen-bond to a hydronium ion weakly correlates with back-and-forth transfer behavior [13, 18, 22]. These findings are presented in detail in Supplementary Note 9.

Supplementary Note 2:

Experimental infrared spectra of aqueous hydrochloric acid solutions

Supplementary Fig. 5a shows experimental IR absorption spectra and Supplementary Fig. 5b shows difference spectra of aqueous HCl solutions at various concentrations. All data is taken directly as published in the literature. The proton continuum band is clearly visible in all difference spectra, albeit with slight differences in shape. In comparison to our calculated difference spectra in Fig. 1b in the main text, the features at 40 THz, 50 THz and 90 THz are more pronounced.

Indeed difference spectra depend on the normalization applied to the underlying HCl solution

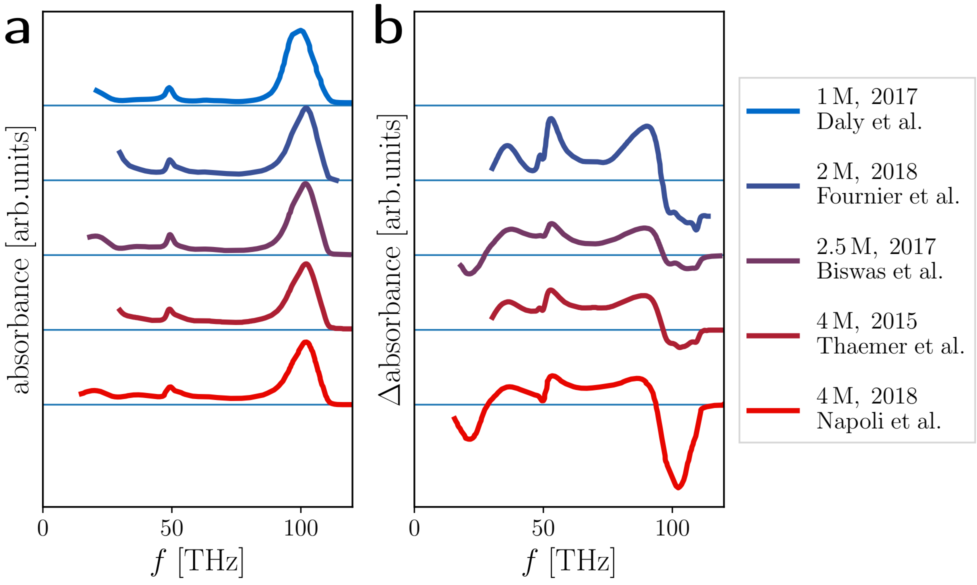

Supplementary Figure 5. Experimental IR absorption spectra (**a**) and corresponding difference spectra (**b**) of aqueous HCl at various concentrations, taken from [11, 24–27]. The data was not published with an absolute scale and is thus scaled arbitrarily. No difference spectrum was published for the spectrum at 1 M [24].

and pure water spectra which is further elucidated in Supplementary Note 3. Normalizations commonly used for spectra calculated from simulations employ sample volume or concentration of water molecules. In experimental setups, however, these parameters are often not reported. Of the four publications we took difference spectra from, only Napoli et al. explained the applied normalization scheme as normalization “to a unit area over the range shown”[11]. In light of these subtleties, comparisons between difference spectra from different sources should be considered with care.

Supplementary Note 3:

Normalization of infrared difference spectra of aqueous hydrochloric acid solutions

The normalization of the difference spectra is subtle and in many studies not well documented as discussed in the previous Supplementary Note 2. In Supplementary Fig. 6, we illustrate how small deviations in the normalization constants can lead to drastic variations among the difference spectra. Throughout this work difference spectra $\omega\Delta\chi''_{c_\mathrm{w}}(\omega)$ are defined as normalized by the water concentration c_w of the aqueous HCl solutions and by the concentration c_w^0 of the reference water spectrum

$$\omega\Delta\chi''_{c_\mathrm{w}}(\omega) = \frac{1}{c_\mathrm{w}}\omega\chi''_\mathrm{solution}(\omega) - \frac{1}{c_\mathrm{w}^0}\omega\chi''_\mathrm{water}(\omega), \tag{19}$$

according to eq. (8) from the methods section. In Supplementary Fig. 6, the difference spectra of the 4 M HCl solution, obtained from ab initio MD simulations in this study, are shown as a blue solid line and compared to difference spectra, that are obtained if instead slightly varied water concentrations for the acid solution c_w^* are used in the normalization. The data illustrates, that even in the moderate range of $c_\mathrm{w}^*/c_\mathrm{w} = 0.8$ to 1.2, some features of the difference spectra, like the dip at the position of the water bend around 1650 cm^{-1} (50 THz), or the features in the OH stretch above 3000 cm^{-1} (90 THz), are significantly changed. These features therefore have to be considered with great care. However, the dominant spectroscopic features, like the continuum band between 2000 cm^{-1} to 3000 cm^{-1} and the peak around 1200 cm^{-1} (35 THz), that are associated with the excess proton dynamics and are at the focus of this study, are modified in height but not in shape and seem therefore very robust with respect to the normalization protocol.

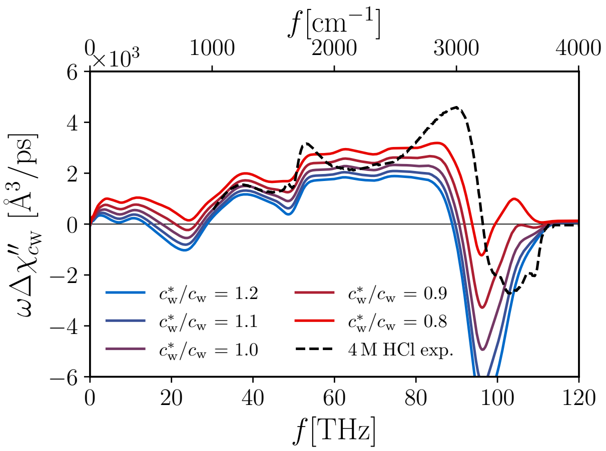

Supplementary Figure 6. Illustration of the effect of variations of the normalization protocol used for difference spectra of the 4 M HCl solution, obtained from ab initio MD simulations. The difference spectra are defined according to eq. (19) and the water concentrations for the acid solution c^*_w are varied (colored solid lines) with ratios reported in the legend. For comparison experimental data for a 4 M HCl solution is shown as a black broken line [27].

Supplementary Note 4:

Spatial correlations

Spatial correlations in liquids can be quantified with radial distribution functions (RDFs), $g_{ab}(r)$, which denote the average density of particles of type a at a relative distance r around particles of type b. The local average density of a relative to b at distance r is given as $\rho_a g_{ab}(r)$ where $\rho_a = N_a/V$ is the global density of a. The RDF is computed from simulation data of two particle species with numbers N_a and N_b that are located at positions $\vec{r}_i$ and $\vec{r}_j$ [28]

$$g_{ab}(r) = \frac{V}{4\pi r^2 N_a N_b} \sum_{i=1}^{N_a} \sum_{j=1}^{N_b} \langle \delta(|\vec{r}_i - \vec{r}_j| - r) \rangle. \tag{20}$$

Average coordination numbers of species a within distance r of particles of species b are obtained by integration over $g_{ab}(r)$

$$N_{ab}(r) = \rho_a \int_0^r dr' 4\pi r'^2 g_{ab}(r'). \tag{21}$$

In this Supplementary Note, the spatial correlations of the excess protons in the HCl solutions are analyzed using RDFs, specifically also the correlations with the chloride ions. Excess protons are identified in the simulation data as the remaining protons after the water molecules are assembled for each oxygen atom with the closest two hydrogen atoms at each time step of the simulation. Here and in Supplementary Note 8 we are not excluding protons close to the chloride ions, in contrast to the analysis of the proton-transfer statistics in the main text, which is described in Supplementary Methods 2. Hydronium ions are defined for each excess proton together with the water molecule of the closest oxygen atom.

4.1. Correlations with the chloride ions

In Supplementary Fig. 7 the RDFs of the chloride ions are shown for the three HCl concentrations with respect to the excess protons as solid lines, all hydrogen nuclei, including excess protons, as broken lines, the oxygen nuclei of the water molecules as dotted lines and the oxygen nuclei of the hydronium ions as dash-dotted lines. For all HCl concentrations, the general features of the RDFs are comparable. But, there are a few concentration-dependent

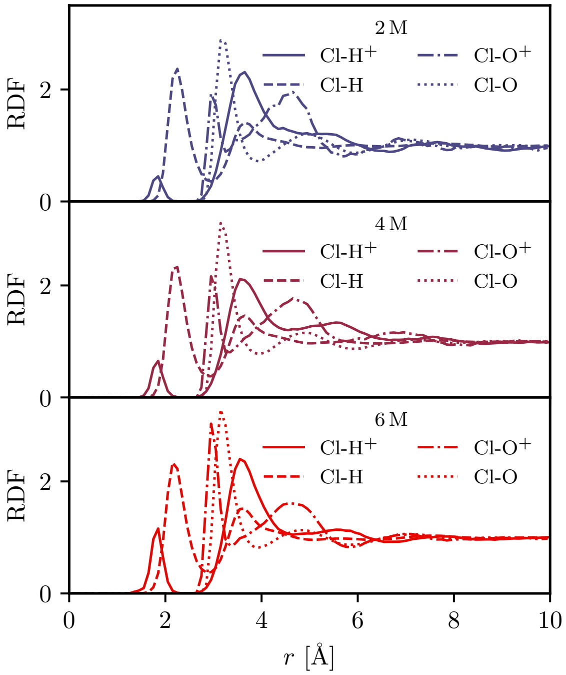

Supplementary Figure 7. Spatial correlations between the chloride ions and other nuclei in terms of radial distribution functions (RDFs) as obtained from ab initio MD simulations of HCl solutions at various concentrations. RDFs are shown for the excess protons (H^+) as solid lines, all hydrogen nuclei (H), including excess protons, as broken lines, the oxygen nuclei of the water molecules (O) as dotted lines and the oxygen nuclei of the hydronium ions (O^+) as dash-dotted lines.

trends. The RDFs of the excess protons around the chloride ions (solid lines) show two dominant peaks, a small one that is located at around $d_{\mathrm{Cl-H^+}} = 1.8\,\text{Å}$ and thereby left of the major peak of the hydrogen nuclei RDFs (broken lines) at around $d_{\mathrm{Cl-H}} = 2.2\,\text{Å}$. This peak is important as it corresponds to excess protons coordinated directly with the chloride ion, i.e. the excess proton sits in between the chloride ion and the oxygen atom of the respective hydronium ion. This contact ion pair is considered an important intermediate in the solvation of HCl [14]. Yet, the excess protons are still part of the hydronium ion, since an even smaller distance of around 1.4 Å would be expected for the covalent bond to the chloride atom, which appears at much higher concentrations than 6 M [14]. The peak height at $d_{\mathrm{Cl-H^+}} = 1.8\,\text{Å}$ (solid lines) clearly increases with HCl concentration in Supplementary Fig. 7, but even for the largest concentration of 6 M it remains smaller than the second and largest peak in the RDFs at about 3.5 Å. This peak is located to the right of the first and

dominant peak in the RDFs of the oxygen nuclei of the hydronium ions (dash-dotted lines) at around $d_{\mathrm{Cl-O^+}} = 3.0\,\text{Å}$, which occurs at a slightly smaller distance than the respective peak of all oxygen nuclei at around $d_{\mathrm{Cl-O}} = 3.1\,\text{Å}$.

All of the presented data is in good agreement with results by Baer et al. [14] from comparable ab initio simulations, who additionally show data for much higher concentrations up to 16 m (16 mol/kg $\simeq$ 11.7 M) and successfully reproduced extended X-ray absorption fine structure (EXAFS) experimental measurements [15]. Specifically, they report $d_{\mathrm{Cl-O^+}} = 2.96\,\text{Å}$ and $d_{\mathrm{Cl-O}} = 3.14\,\text{Å}$ in excellent agreement with experimental EXAFS data [15]. Data comparing various exchange-correlation functionals used in the ab initio simulation also consistently reproduced the results presented in Supplementary Fig. 7 with no significant dependence on the type of functional (except for the PBE functional), albeit with large statistical uncertainties in some data [18].

When interpreting the RDFs, the actual densities of the species need to be put into perspective. The global densities of chloride ions, excess protons and hydronium ions are equal by definition. However, for the 2 M HCl solution data set, the ratio of water molecules to chloride ions or respectively excess protons is about 24.8 and drops to 11.2 for the 4 M and 6.5 for the 6 M data sets. These numbers would have to be divided by two, assuming that water molecules are equally and exclusively solvating all chloride ions and excess protons. It is therefore evident, that for the 6 M solution, hydronium ions are necessarily residing already in the first hydration shell of the chloride ions. More precisely, the average coordination numbers around the chloride ions are obtained by integration over the first peak of each RDF in Supplementary Fig. 7 and use of eq. (21). The obtained coordination numbers are reported in Supplementary Tab. III and are generally in good agreement with previous simulation and experimental results [14, 15]. The average number of hydronium ions in the first hydration shell of any chloride ion increases from 0.13 for 2 M to 0.52 for 6 M, see third column in Supplementary Tab. III. Previously, Baer et al. [14] interpreted this significant increase in the coordination number as an increase of contact ion pairs, where the excess proton is shared between the hydronium oxygen and the chloride ion. However, the coordination number of the excess protons around chloride ions does not increase as much, only from 0.007 for 2 M to 0.054 for 6 M, see first column in Supplementary Tab. III. Therefore, the excess protons of the hydronium ions in the first solvation shell of the chloride ions mostly point away from chloride ions and are predominantly coordinated with other water molecules, rather than

Supplementary Table III. Average coordination numbers N_{ab} around the chloride ions as obtained from the first peak of the RDFs in Supplementary Fig. 7 and eq. (21). Errors of the simulation data are estimated from the resolution of the RDFs.

	Cl-H$^+$ ($r < 2.5$ Å)	Cl-H ($r < 3.0$ Å)	Cl-O$^+$ ($r < 3.5$ Å)	Cl-O ($r < 3.5$ Å)
2 M	0.007 ± 0.001	5.64 ± 0.07	0.13 ± 0.01	4.8 ± 0.1
4 M	0.020 ± 0.001	5.42 ± 0.07	0.26 ± 0.01	4.6 ± 0.1
6 M	0.054 ± 0.001	5.12 ± 0.07	0.51 ± 0.01	4.2 ± 0.1
6 M, MOLOPT	0.091 ± 0.001	5.0 ± 0.1	0.56 ± 0.02	4.0 ± 0.2
6 M, TZV2P	0.115 ± 0.001	5.0 ± 0.2	0.63 ± 0.02	4.0 ± 0.2
2.5 m [14]			0.17	5.82
6 m [14]			0.42	5.21
10 m [14]			0.71	4.67
16 m [14]			1.05	3.99
6 m [14, 15]			0.7 ± 0.2	5.1 ± 0.5
10 m [14, 15]			1.0 ± 0.3	4.8 ± 0.5
16 m [14, 15]			1.6 ± 0.3	4.2 ± 0.5

with chloride ions. However, it is noteworthy that the chloride-excess-proton coordination is affected, when a different basis set is considered in the simulations, which is shown below in subsection 4.3 for simulations at 6 M. In particular, an increase of the coordination of the excess protons with the chloride ions is observed when considering the MOLOPT or TZV2P basis sets. Therefore, while the above results have to be considered with care when comparing to experimental data and additional analysis with more elaborate basis sets may be useful, the analysis performed in this study is self-consistent for the MOLOPT-SR basis set.

Taken together, this data indicates that the excess protons in aqueous HCl solutions mostly reside inside hydronium ions within our definition, and additionally stay away from the chloride ions, i.e. are located between two oxygen atoms and not between a chloride and an oxygen atom. This holds true even for moderately high concentrations up to 6 M considered in this study, but necessarily breaks down at very high concentrations when the water becomes saturated with HCl [14]. The data confirms the high solubility of excess protons in water, due

to energetic but also entropic effects [17, 29]. Furthermore, it justifies the focus on proton transfer between water molecules and the excess-proton selection and identification scheme in Supplementary Methods 2 to analyze the excess-proton spectral signatures of the HCl solutions. Note, that already the linear trend of the HCl difference spectra with concentration shown in Fig. 1d in the main text indicates that in the range up to 6 M considered in the ab initio MD simulations in this study, the effect of the chloride ions on the excess-proton spectra and dynamics is negligible. Similarly, Napoli et al. [11] previously showed that IR spectra of excess protons coordinated with chloride ions show much weaker spectral signatures as compared to the average spectra of all the excess protons.

4.2. Correlations between oxygen and hydrogen atoms

In Supplementary Fig. 8a the spatial correlations of the hydronium ions and in Supplementary Fig. 8b of the excess protons themselves are discussed. Comparable results for aqueous HCl solutions were previously shown for various exchange-correlation functionals used in ab initio simulations [18] and for different self-consistent iterative multistate empirical valence bond (SCI-MS-EVB) simulations [16, 17].

In Supplementary Fig. 8a the RDFs of the oxygen nuclei of the hydronium ions are shown with respect to the oxygen nuclei of other hydronium ions (O^+) as solid lines, for the oxygen nuclei of the water molecules (O) as broken lines, for the the excess protons (H^+) as dash-dotted lines and all hydrogen nuclei (H), including excess protons, as dotted lines. Next to the trivial peaks of the dash-dotted as well as the dotted lines at below 1.0 Å, belonging to the hydrogen nuclei which are part of the hydronium ions itself, a clear peak of the broken lines at $d_{O^+-O} = 2.5$ Å indicates the water oxygen nuclei in the first hydration shell, each of which are candidates to form a transient $H_5O_2{}^+$ complex, i.e. the special pair, together with the hydronium ion. This peak is roughly consistent with the most probable oxygen-oxygen distance of the transient $H_5O_2{}^+$ complex, that is seen in the free energy in Fig. 4a in the main text. A second, much smaller, peak at about 4.2 Å indicates the water molecules in the second hydration shell of the hydronium ion. Boths peaks at about $d_{O^+-O} = 2.5$ Å and at 4.2 Å are clearly shown in data for various exchange-correlation functionals [18], as well as for SCI-MS-EVB simulations [16, 17]. Beyond that, a decomposition of the first peak into three components was used to support the picture of an asymmetric Eigen state [9].

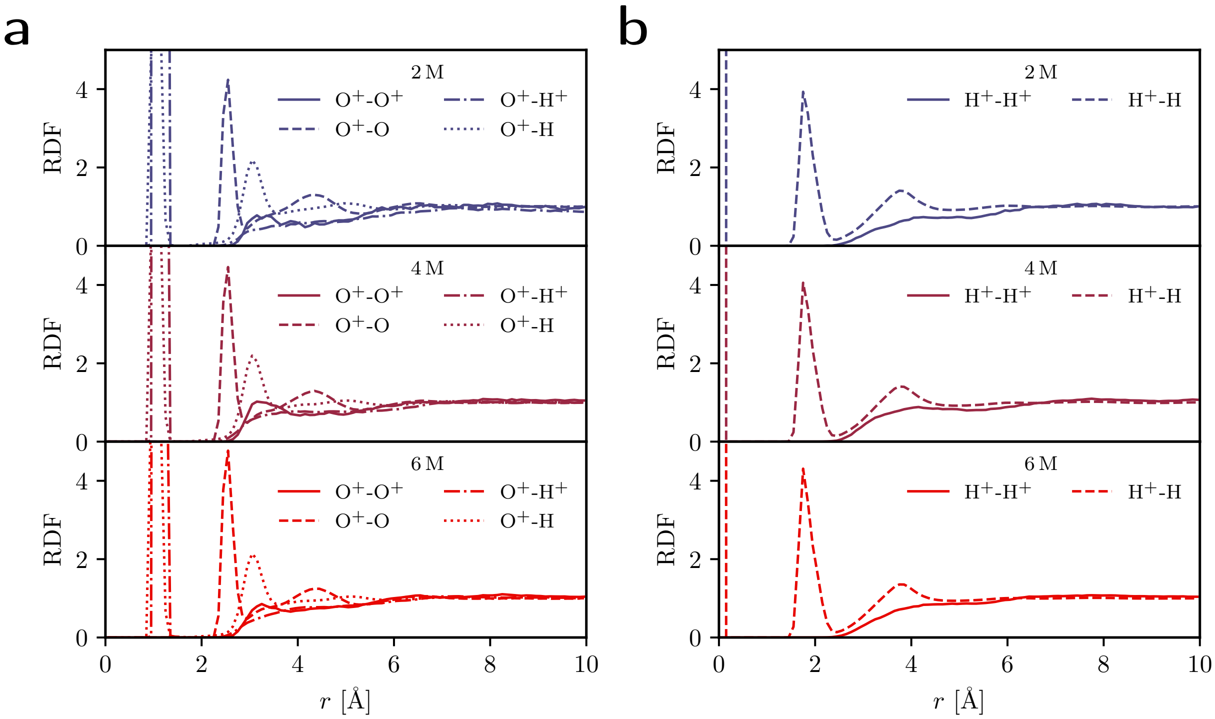

Supplementary Figure 8. Spatial correlations between the oxygen atoms of hydronium ions (a) and excess protons (b) with respect to other nuclei in terms of radial distribution functions (RDFs) as obtained from ab initio MD simulations of HCl solutions at various concentrations. **a:** RDFs are shown for the oxygen nuclei of the hydronium ions (O^+) as solid lines, for the oxygen nuclei of the water molecules (O) as broken lines, for the the excess protons (H^+) as dash-dotted lines and all hydrogen nuclei (H), including excess protons, as dotted lines. **b:** RDFs are shown for the excess protons (H^+) as solid lines and all hydrogen nuclei (H), including excess protons, as broken lines.

Each peak is accompanied by peaks of the dotted lines at slightly larger distances, which indicate the hydrogen atoms belonging to the water molecules in the respective hydration shells. Additionally, the dotted lines show a weak and broad shoulder below roughly 2.5 Å, the origin of which is further discussed in Supplementary Note 9. A slight relative maximum of the solid lines at $d_{O^+-O^+} = 3.00$ Å but with a magnitude below one indicates a metastable structure between two hydronium ions. However, note that there is no such signature in the dash-dotted lines, i.e. no apparent correlation with the excess proton of the nearest hydronium ion. The peak at $d_{O^+-O^+} = 3.00$ Å was also observed by Calio et al. [17] and at $d_{O^+-O^+} = 3.20$ Å by Xu et al. [16].

In Supplementary Fig. 8b the RDFs of the excess protons are shown with respect to the

other excess protons (H^+) as solid lines and all hydrogen nuclei (H), including excess protons, as broken lines. The main peak of the broken lines at $d_{H^+-H} = 1.8$ Å indicates the mean distances between hydrogen atoms within the same hydronium ion. The peak shows a shoulder at around 2.1 Å possibly related to spatial correlations between the excess proton and two hydrogen atoms in the neighboring water molecule of the transient $H_5O_2{}^+$ complex, i.e. of the special pair. The second peak of the broken lines at 3.8 Å is related to other water molecules in the first hydration shell, that form hydrogen bonds (HBs) with the hydronium ion to which the excess proton is assigned. No spatial correlations are apparent in the RDFs between excess protons, shown as solid lines. In contrast to that, Xu et al. [16] observed a slight peak at $d_{H^+-H^+} = 4.20$ Å, indicative of spatial correlations between two excess protons. In summary, the RDFs of the hydronium ions as well as of the excess protons presented in Supplementary Figs. 8a and b indicate no spatial correlations between excess protons or hydronium ions and other excess protons. While there appears a slight relative maximum in the RDF between hydronium ions itself, this may simply be related to close-packing effects in the liquid.

4.3. Results for different basis sets

To address the quality of the simulations for the chosen MOLOPT-SR basis set, additional simulations of HCl solutions at 6 M with the DZVP-MOLOPT-GTH and TZV2P-GTH basis sets are performed [30, 31]. For both cases the chloride ions are modeled including diffuse functions by using the aug-DZVP-GTH and aug-TZV2P-GTH basis sets, respectively. Otherwise the setups are equivalent to the MOLOPT-SR simulations and each simulation is run for 28 ps under NVT condition. The spatial correlations in each data set are then compared by computing RDFs, the results of which are presented in Supplementary Figs. 9a–e. The correlations of the oxygen atoms and the hydrogen atoms among each other, that are most important for the solvation structure of the excess protons and therefore the focus of this study as discussed above, show no significant dependence on the choice of basis set as is seen from the near-perfect agreement of the RDF curves throughout Supplementary Figs. 9c–e. Regarding the chloride ions, some differences in the RDFs in Supplementary Figs. 9a and b are clearly discernible though. Most importantly, the first major peaks in the RDFs between chloride ions and oxygen atoms of hydronium ions (solid lines in Supplementary Fig. 9a) as

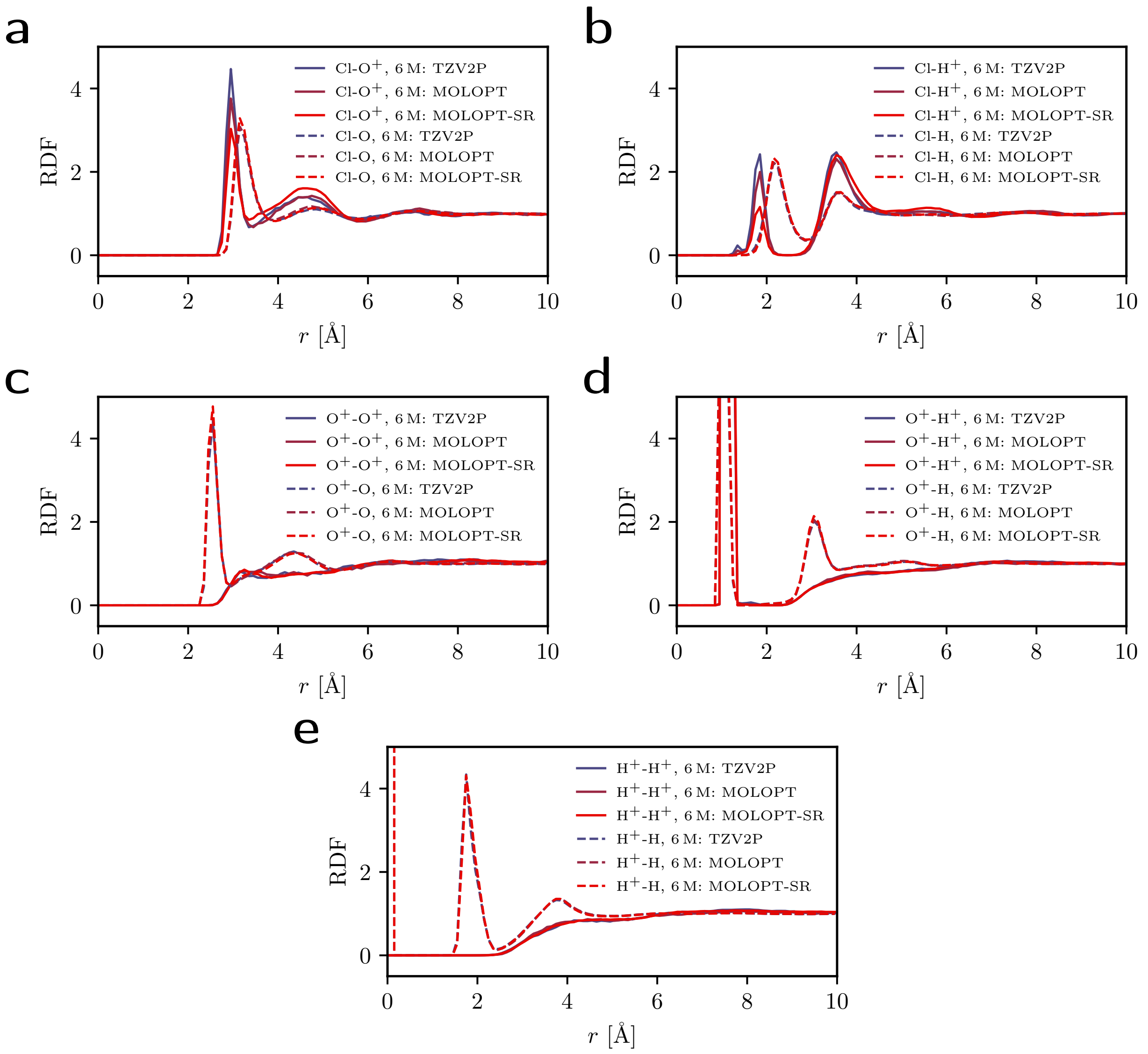

Supplementary Figure 9. Spatial correlations between the chloride ions and oxygen atoms (a), chloride ions and hydrogen atoms (b), oxygen atoms of hydronium ions and oxygen atoms (c) or hydrogen atoms (d), and excess protons (e) with respect to other nuclei in terms of radial distribution functions (RDFs) as obtained from ab initio MD simulations of HCl solutions at 6 M for various basis sets considered in the simulation. Data obtained for the TZV2P basis set is shown in blue, for the MOLOPT basis set in purple and for the MOLOPT-SR basis set in red. **a,c:** RDFs are shown for the oxygen nuclei of the hydronium ions (O^+) as solid lines, for the oxygen nuclei of the water molecules (O) as broken lines. **b,d,e:** RDFs are shown for the excess protons (H^+) as solid lines and all hydrogen nuclei (H), including excess protons, as broken lines.

well as between chloride ions and excess protons (solid lines in Supplementary Fig. 9b) increase when the MOLOPT or TZV2P basis sets are used. Additionally, a second much smaller peak is visible at a closer distance for both simulations with the MOLOPT and TZV2P basis sets (solid blue and purple lines in Supplementary Fig. 9b), indicating that contact pairs between chloride ions and excess protons appear rarely throughout the simulations. This means that the role of the chloride spectator, that is argued in Supplementary Note 4.1 to be of minor importance, may be underestimated in simulations using the MOLOPT-SR basis set. In Supplementary Note 4.1 the coordination number of the excess protons around chloride ions at 6 M was found to be 0.054 from integration over the first major peak of the RDF up to 2.5 Å (solid red line in Supplementary Fig. 9b and first column in Supplementary Tab. 4.1). When considering the MOLOPT basis set, this value increases to 0.091 and for the TZV2P basis set to 0.115, meaning that on average each excess proton is for 9% or respectively 12% of the time coordinated with a chloride ion as the second nearest neighbour (or rarely even as the nearest neighbor) in a configuration that is not well described by a transient $H_5O_2{}^+$ complex which forms the basis of the analysis performed in the main text.

Supplementary Note 5:

Decomposition of infrared spectra of transient $H_5O_2^+$ complexes

To allow for a decomposition of IR spectra of transient $H_5O_2^+$ complexes into contributions perpendicular and parallel to the connecting axis of the water oxygens, the dynamics of the transient $H_5O_2^+$ complexes are described in a comoving internal coordinate system as introduced in the main text and shown in Supplementary Fig. 10c. The x-axis is defined as the axis connecting both oxygen atoms (in the following referred to as O-O axis), the coordinate origin being located in the middle between the oxygens. The yz-plane lies perpendicular to that axis. A transformation to this coordinate system thus involves a time-dependent translation and rotation of the trajectory $\mathbf{r}_i(t)$ in the laboratory frame of each atom inside a $H_5O_2^+$ complex and can be described by

$$\mathbf{r'}_i(t) = \mathbf{M}_{\phi(t),\theta(t)}\,[\mathbf{r}_i(t) - \mathbf{r}_0(t)], \tag{22}$$

where $\mathbf{r}_0(t)$ is the trajectory of the oxygen-oxygen midpoint and $\mathbf{M}_{\phi(t),\theta(t)}$ is a rotation matrix setting both oxygen atoms on the x-axis. The coordinate transform is illustrated in Supplementary Fig. 10. These operations do of course have an influence on the resulting spectra, which is discussed in Supplementary Note 6.

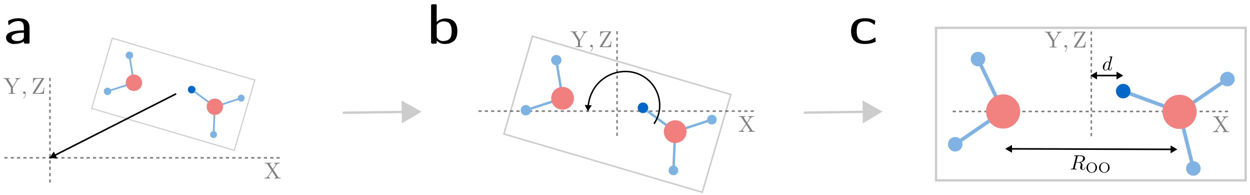

Supplementary Figure 10. Illustration of the coordinate transform eq. (22) from the laboratory frame to the internal coordinate system of the transient $H_5O_2^+$ complexes, allowing for a distinction of movements perpendicular and parallel to the connecting axis between the water oxygens (referred to as O-O axis). **a:** First the oxygen-oxygen midpoint $\mathbf{r}_0(t)$ is subtracted from the coordinates of the atoms of the transient subsystem $\mathbf{r}_i(t)$. **b:** Then the atoms are rotated, such that both oxygen atoms lie on the x-axis. **c:** In the resulting internal coordinate system the variable d is defined as the projection of the excess proton location onto the x-axis. R_{OO} is the distance between the two oxygen atoms.

The internal coordinate system further defines d, the projection of the excess proton location onto the O-O axis, and R_{OO}, the distance between the two oxygen atoms according to

$$R_{\mathrm{OO}}(t) = |x'_{\mathrm{O}_1}(t) - x'_{\mathrm{O}_2}(t)|$$
$$d(t) = x'_{\mathrm{H}+}(t).$$

Using such an internal coordinate system, we can decompose the spectra of protons and water molecules inside the transient $H_5O_2^+$ complexes as well as their cross-correlation spectra into their contribution along and perpendicular to the O-O axis, respectively.

For that purpose, we extract the trajectories of the closest two water molecules to each previously extracted excess-proton trajectory (see Supplementary Methods 2), resulting in trajectories of transient $H_5O_2^+$ complexes. Since the water molecules involve valence electrons, we use the localized-Wannier-center trajectory data for this, resulting in trajectories with a slightly lower time resolution of 4 fs than for the nuclei-only data. In order to be able to decompose the trajectories into parts perpendicular and parallel to the O-O axis, we describe the multidimensional $H_5O_2^+$ trajectories using the internal coordinate system introduced above and calculate spectra accordingly.

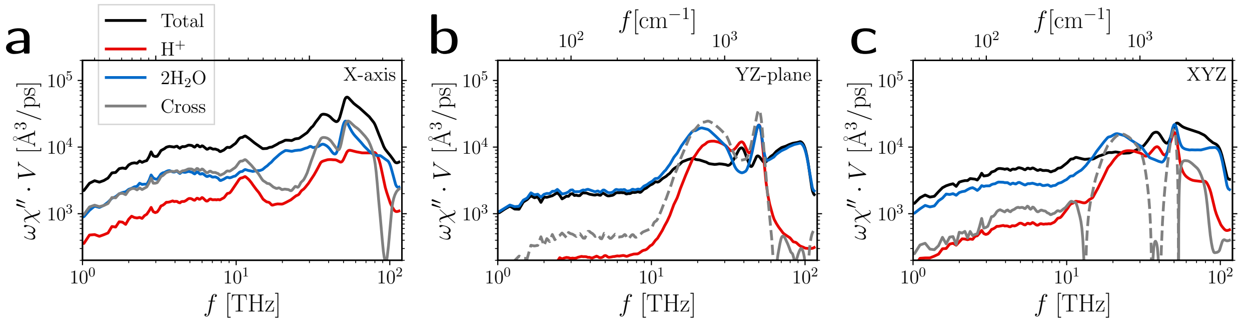

Supplementary Figure 11. Absorption spectra including nuclei and electrons of transient $H_5O_2^+$ complexes consisting of their excess protons (H^+, red solid lines), their closest two water molecules (2 H_2O, blue solid lines) and cross correlations (Cross, gray solid and broken lines) parallel to the O-O axis (**a**) and perpendicular to it, i.e. in the yz-plane of the local coordinate system (**b**). The isotropic spectrum is shown in **c**. Broken lines indicate negative values. Note that the spectra are averaged over the spatial dimensions.

In Supplementary Fig. 11 the resulting spectra are shown, separated into the contributions along (Supplementary Fig. 11A) and perpendicular to the O-O axis (Supplementary Fig. 11b)

and each averaged over their spatial dimensions. The excess-proton spectrum perpendicular to the O-O axis (Supplementary Fig. 11b, red solid lines) clearly dominates the isotropic excess-proton spectrum (Supplementary Fig. 11c, red solid line). Including cross correlations, defined as the difference between the total spectrum and the sum of the proton and water contributions, a different picture emerges. In Supplementary Fig. 11a and b the cross correlations between the excess proton and its two closest water molecules are plotted in gray. The cross correlations between proton and water molecules are of the same order of magnitude and of similar shape as the proton spectrum itself and almost entirely positive along the x-axis and negative along the yz-plane, meaning that the water polarization is to some degree amplifying the proton's polarization dynamics along the O-O axis, whereas they cancel perpendicular to the O-O axis. This phenomenon was similarly observed for isolated $H_5O_2{}^+$ complexes [32]. Also, Sauer and Döbler [33] found in an MD study of $H_5O_2{}^+$ that "[...] the O-H^+-$O_{Y,Z}$ bends (perpendicular to the O-O axis) have vanishing IR intensities and should not be seen in the spectra [...]".

The analysis presented in this Supplementary Note shows, that the IR spectra of $H_5O_2{}^+$ complexes in HCl solutions dominantly represent the motion of the excess proton along the O-O axis. This motivates the projection of the protons' movement onto the O-O axis, d, that is used for the decomposition in the main text.

Supplementary Note 6:

Spectral signature resulting from change of coordinate system

As presented in Supplementary Note 5, the dynamics of the transient ${H_5O_2}^+$ complexes, including the excess protons, is described in the comoving internal coordinate systems. To estimate the effect of this coordinate transformation on the spectrum of protons and water molecules in the ${H_5O_2}^+$ complexes, we compare the decomposed spectra of these complexes in the original coordinate system to those after translation and subsequent rotation, respectively. Figure 12 shows a comparison between the spectrum of ${H_5O_2}^+$ complexes in their original coordinate system and translated according to $\mathbf{r}_{i,\text{trans}}(t) = \mathbf{r}_i(t) - \mathbf{r}_0(t)$ (compare eq. (22)). The translation does not have any effect on the spectrum of the two water molecules because they are not charged and the dipole moment of a neutral system does not change

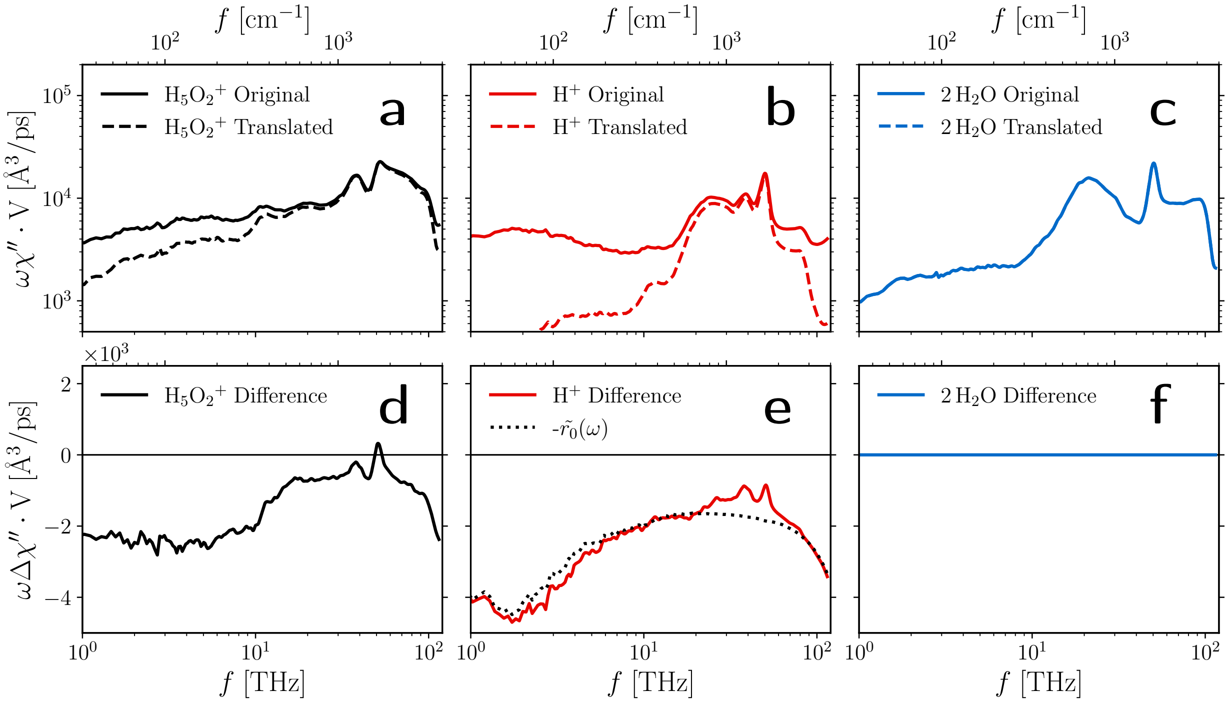

Supplementary Figure 12. **a–c:** Averaged non-normalized absorption spectra including nuclei and electrons of transient ${H_5O_2}^+$ complexes in their original coordinate system (solid) and translated such that the coordinate origin is the midpoint between the oxygens (broken). **d–f:** The differences between the spectra above. A dotted line in e shows the spectrum of the movement of the oxygen midpoint $\mathbf{r}_0(t)$.

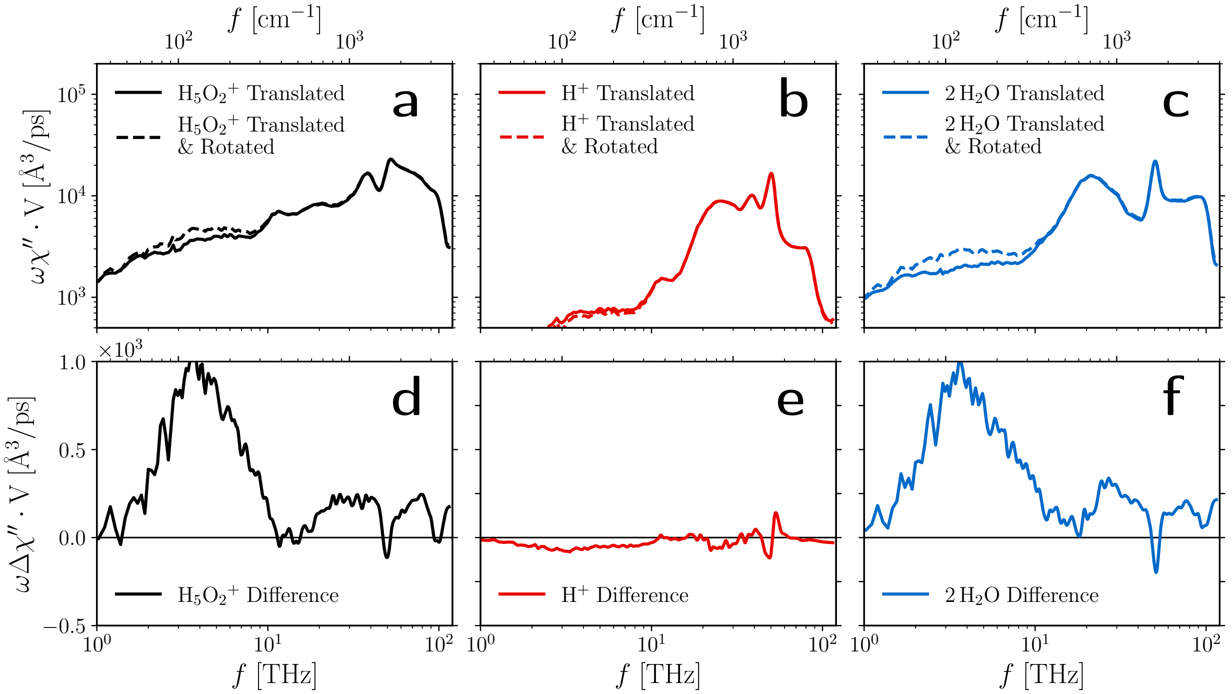

Supplementary Figure 13. **a–c:** Non-normalized absorption spectra including nuclei and electrons of transient $H_5O_2^+$ complexes that are translated but in their original orientation (solid) and additionally rotated such that the oxygen atoms lie on the O-O axis (broken). **d–f:** The differences between the spectra above.

upon translation. The excess proton, however, has a charge. Thus, its dipole moment is affected by this translation. The effect can be approximated by the spectrum of all oxygen midpoint trajectories $\mathbf{r}_0(t)$ of the $H_5O_2^+$ complexes using a charge of 1 e, which is shown in Supplementary Fig. 12e as a dotted line. While the translation leads to significant change in the proton spectrum at low and high frequencies, in the frequency range between 20 THz to 80 THz the effect of the translation is mostly negligible. Obviously, the error in the projected spectrum in Supplementary Fig. 12b is not transferred to the single-proton spectrum projected along the oxygen-oxygen distance coordinate d, that we show in Fig. 3c in the main text.

In contrast to the translation, the rotation mostly affects the water spectrum, because the translation due to rotation is larger for atoms further away from the rotation center. This can be seen in Supplementary Fig. 13 which shows a comparison between the spectrum of $H_5O_2^+$ complexes that were translated as shown in Supplementary Fig. 12 and those that

were additionally rotated according to $\mathbf{r'}_i(t) = \mathbf{M}_{\phi(t),\theta(t)}\,\mathbf{r}_{i,\mathrm{trans}}(t)$ (compare eq. (22)). The proton spectrum is barely affected and the effect on the water spectrum is limited to low frequencies.

Since both translation and rotation have at most a minor effect on the transient $H_5O_2{}^+$ spectrum in the relevant frequency range, our findings from the decomposition into x- and yz-contribution in Supplementary Note 5 for transient $H_5O_2{}^+$ complexes also apply to the lab frame.

Supplementary Note 7:

Distribution of proton-transfer waiting times

The mean proton-transfer rate is at the heart of research on excess protons solvated in water, as it is the relevant microscopic time scale that determines the macroscopic large diffusion obtained by the Grotthuss process. As outlined in the main text, the mean transfer rate corresponds to the waiting time of a stochastic barrier-crossing process, that is not to be confused with the transfer-path time of the actual transition over the barrier. The waiting-time distributions that are also shown in Fig. 5c in the main text are discussed in this Supplementary Note and are compared to previous results from literature.

Agmon [34] gives a summary of early experimental results, suggesting a mean proton-transfer time of 1.5 ps obtained in NMR studies [35], which is believed to be correlated with the hydrogen bond rearrangement and water reorientation dynamics on the time scales of 1 ps to 2 ps determined from a number of other experiments. These time scales seem to contrast more recent experimental results from 2D IR spectroscopy, that report interconversion between different proton hydration structures, i.e. Eigen and Zundel-like structures, on time scales of around 100 fs and less [20, 36, 37]. On the other hand, Carpenter et al. [38] write, "the hydrated proton bend displays fast vibrational relaxation and spectral diffusion timescales of $200 - 300$ fs, however, the transient absorption anisotropy decays on a remarkably long 2.5 ps timescale, which matches the timescale for hydrogen bond reorganization in liquid water". Arguing that the latter would be an upper bound, they infer "the transfer of excess protons in water [...] is an activated process with a timescale of $1 - 2$ ps." Kundu et al. [20] confirm that "during the lifetime of the $H_5O_2^+$ motif, that is on the order of 1 ps, the proton undergoes fluctuating large-amplitude motions exploring essentially all possible positions between the flanking water molecules". Yuan et al. [39] measure a concentration-dependent 'proton hopping time' in HCl solutions using 2D IR chemical exchange spectroscopy with a methyl thiocyanate probe and extrapolate a time of 1.6 ps for the dilute limit.

It transpires, that two time scales determine the distribution of proton-transfer waiting times, which was confirmed in various simulation studies and interpreted as stemming from either back-and-forth or uni-directional proton transfer, respectively, in the literature sometimes referred to as 'reversible' and 'irreversible' proton transfer. Napoli et al. [11] point out that

Supplementary Table IV. Collection of proton-transfer time scales reported in the literature.

	method	conc. [M]	T [K]		time
Meiboom [35]	NMR exp.				1.5 ps
Woutersen and Bakker [36]	2D IR exp.	5	300		<0.1 ps
Dahms et al. [37]	2D IR exp.	<1	300		<0.1 ps
Thämer et al. [27]	2D IR exp.	4	300		>0.48 ps
Carpenter et al. [38]	2D IR exp.	2	300		<2.5 ps
Kundu et al. [20]	2D IR exp.	1	300		<0.1 ps
					1 ps
Yuan et al. [39]	2D IR chemical	dillute	300		1.6 ps
	exchange exp.	limit			
Fischer et al. [40]	DFT CPMD	1.7	300	bi-directional	0.5 ps
Fischer and Gunlycke [18]	DFT CPMD	1.7	300	uni-directional	2.5 ps
Calio et al. [17]	MS-EVB	$0.43-3.26$	300		$0.4-0.5$ ps
					$1.2-2.3$ ps
Calio et al. [9]	MS-EVB,	$0.22-0.43$	300		$10-17$ fs
	DFT BOMD				$0.3-0.5$ ps
					$2.3-3.2$ ps
	+ nuc.-quantum				$12-15$ fs
	effects				$0.83-0.27$ ps
					1.4 ps
Arntsen et al. [19]	DFT BOMD		300	bi-directional	0.184 ps
				uni-directional	1.69 ps
Roy et al. [41]	DFT BOMD	2	300		$1-2$ ps
	DFT BOMD	8	300		$2-4$ ps

while they find a frequency-correlation time of (1.4 ± 0.3) ps "corresponding to the pump ($3150\,\mathrm{cm}^{-1}$) and probe ($1760\,\mathrm{cm}^{-1}$) frequencies used in [27]", the auto-correlation of the proton asymmetry actually decays on time scales of less than 100 fs with a second slower component of (0.8 ± 0.1) ps. Fischer et al. [40] find the time scale of proton 'hopping' to be around 0.5 ps, including back-and-forth events, and deduce a time scale of 2.5 ps for uni-directional events in a later study [18]. Calio et al. [17] extract two timescales of about 400 fs to 500 fs and 1.3 ps to 2.3 ps for the concentrations 0.43 M to 3.26 M from fits to the long-lived anisotropy decays, which the authors argue "can correlate experimental time constants to irreversible proton transfer". In a follow-up study Calio et al. [9] report three timescales of about $10 - 17$ fs, $320 - 490$ fs and $2.3 - 3.2$ ps for the concentrations 0.22 M to 0.43 M from fits to the anisotropy decay of the excess-proton dynamics projected on the axis of the two closest oxygen atoms, with a slight acceleration to the values $12 - 15$ fs, $83 - 270$ fs and 1.4 ps when nuclear-quantum effects are considered. Roy et al. [41] find a time scale of $1 - 2$ ps for uni-directional proton transfer between two water molecules in 2 M HCl solutions employing two-dimensional transition state theory and Marcus theory of ion pairing. This number increases significantly to $2 - 4$ ps in 8 M HCl solutions. Arntsen et al. [19] report time constants of the excess-proton identity auto-correlation function, which is elaborated on further below. They find a values of 184 fs, but after eliminating back-and-forth events from the data the time scale increases to 1.69 ps. Furthermore, a couple of studies point out that the long time scale of proton transport increases significantly with concentration [29, 41].

A summary of the reported values is given in Supplementary Tab. IV. It transpires that the separation in back-and-forth and uni-directional events is important to distinguish two time scales in the broad distributions of proton-transfer times. For stochastic barrier-crossing processes of highly inertial or non-Markovian coordinates and furthermore for low energy barriers, it is well established that barrier-crossing events exhibit large numbers of subsequent back-and-forth events, due to the slow dissipation of the energy required for the initial barrier-crossing event [32, 41–45]. Such events appear especially important with regard to proton-transfer processes and their spectral signatures.

The proton-transfer waiting time distributions of the excess-protons in aqueous HCl solutions at various concentrations, reported in the main text in Fig. 5c, are plotted in Supplementary Fig. 14a on a double logarithmic scale in order to investigate the long time behavior. The distributions do decay in the picosecond range with a clear concentration dependence, i.e.

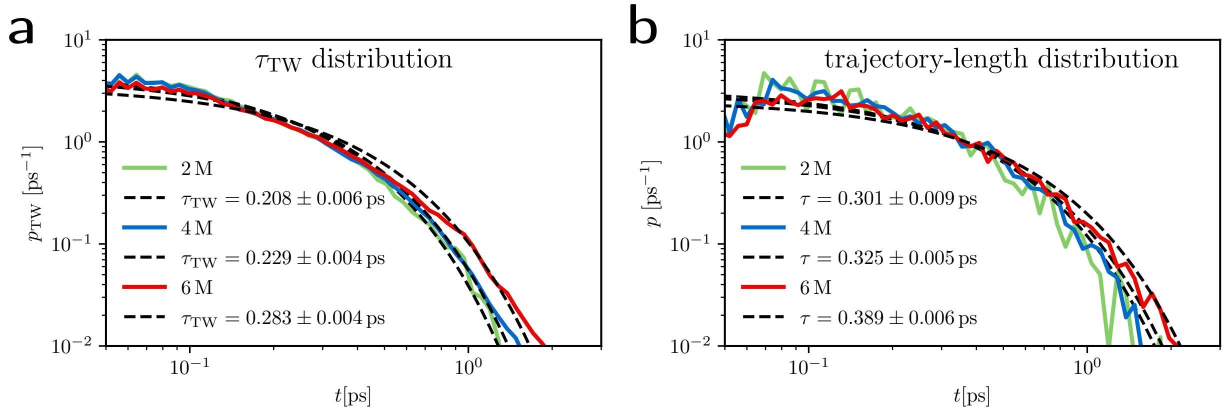

Supplementary Figure 14. Distributions of proton-transfer waiting times (a) and of the life times of the transient $H_5O_2{}^+$ clusters, i.e. the trajectory lengths, (b) in HCl solutions at various concentrations as obtained from ab initio MD simulations. The same data as in a is also shown in Fig. 5c in the main text. For comparison, single exponential decays, $p(t) = \exp(-t/\tau)/\tau$, using the mean of the distributions τ reported in the legend, are plotted as black broken lines.

the distributions have a longer tail for higher concentrations. Still, a single exponential fit using the mean of the distribution τ_{TW} as the decay time scale, $p_{\mathrm{TW}}(t) = \exp(-t/\tau_{\mathrm{TW}})/\tau_{\mathrm{TW}}$, is sufficient to describe the long time tail of the distributions. However, since the excess protons are tracked only in the transient $H_5O_2{}^+$ cluster, the transfer-waiting times can only capture proton transfer during the life times of these $H_5O_2{}^+$ clusters, i.e. the lengths of the trajectories, that are defined according to the procedure that is detailed in Supplementary Methods 2. The distributions of trajectory lengths are given in Supplementary Fig. 14b. They are again compared to single exponential functions, $p(t) = \exp(-t/\tau)/\tau$, shown as black broken lines, with the mean values of the distributions as the decay constants τ. For all three concentrations the mean values are about 1.5 times larger than the respective waiting-times reported in Supplementary Fig. 14a, indicating that on average one to two transfers occur during the life time of a transient $H_5O_2{}^+$ cluster.

Identity auto-correlation functions

To describe the long time scales of excess-proton diffusion observed in the anisotropy decay of 2D IR experiments [27, 38], correlation times of the excess-proton identity have proven useful. Inspired by previous work [9, 19, 46], we computed auto-correlation functions of

the excess-proton and hydronium-oxygen identities from joint trajectories of the excess protons as also prepared for the analysis of the long-time diffusion properties described in Supplementary Note 8. Following this protocol, the excess protons are given as the remaining protons after the water molecules are assembled for each oxygen atom with the closest two hydrogen atoms at each time step of the simulation. Hydronium ions are defined by an excess proton together with the water molecule of the closest oxygen atom. Therefore, at each time step a total number of excess protons N_{H^+}, as well as hydronium ions, equivalent to the number of chloride ions in the simulation, is obtained. The trajectories are then stitched together to give N_{H^+} trajectories, each of the length of the whole simulation, for the excess protons and hydronium oxygens, respectively. Following Arntsen et al. [19], rapid back-and-forth fluctuation of hydronium-oxygen identities is 'filtered' from the trajectories by the following procedure. Whenever along a trajectory the identity changes from one oxygen atom to another, we check whether the identity returns to the original nucleus within 0.5 ps. If it returns without passing to a third nucleus in between, the identity remains with the original nucleus as if the identity did not change throughout this time. For the excess-proton identities this criterion does not suffice since the identity often fluctuates between three candidates within one hydronium ion. Rather the same procedure as also detailed in Supplementary Note 8 and similar to Calio et al. [9] is used to 'filter' the rapid back-and-forth fluctuation of excess-proton identities, i.e. the 'special pair dance': The candidate proton that either performs the next transfer to another water molecule or is the next to be identified as an excess proton while neighboring a chloride atom, remains the excess proton. These procedures define sets of trajectories from which the fast identity fluctuations are 'filtered'. The identity auto-correlation functions are then calculated for excess-proton and hydronium-oxygen identities on both sets of trajectories, 'filtered' and 'unfiltered'.

We define the identity auto-correlation function as

$$c(t) = \frac{\langle h(t)h(0)\rangle - \langle h\rangle^2}{\langle h\rangle - \langle h\rangle^2}, \tag{23}$$

where $h(t)$ is one if an excess proton or hydronium oxygen atom has the same identity, i.e. is the same nucleus, as at $t = 0$, otherwise $h(t)$ is zero. In our definition the identity auto-correlation function is designed to reach unity for $t = 0$ and to decay to zero for long times and is thereby slightly different compared to previous work [19, 46, 47]. Additionally,

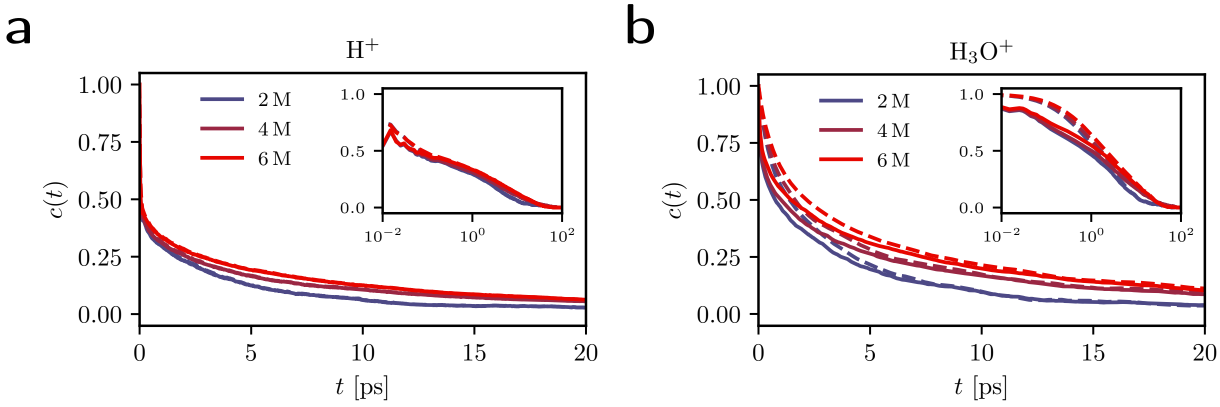

Supplementary Figure 15. Identity auto-correlation functions eq. (23) of the excess protons (a) and hydronium oxygens (b) obtained from ab initio MD simulations of HCl solutions at various concentrations. The correlations functions are computed from unfiltered (solid colored lines) and filtered trajectories (broken colored lines), see text for details. The data is shown on a logarithmic time axis in the insets.

the continuous identity auto-correlation function is given as [9, 19]

$$C(t) = \frac{\langle H(t)H(0)\rangle}{\langle H\rangle}, \tag{24}$$

where $H(t)$ is one as long as an excess proton or hydronium oxygen atom continuously has the same identity for the entire time interval $[0, t]$ and zero otherwise.

The excess-proton and hydronium-oxygen identity auto correlations obtained from our data according to eq. (23) are given in Supplementary Figs. 15a and b with the same data shown on a logarithmic time axis in the insets. Auto-correlation functions of the filtered trajectories are shown as broken colored lines for the three HCl solutions at various concentrations while the auto-correlation functions of the unfiltered trajectories are shown as solid colored lines. The curves decay on multiple time scales. All show remarkable long-time behavior beyond several picoseconds, indicating that proton as well as hydronium identity is correlated over very long times scales, which occurs from looping of identities over several different nuclei [46]. As expected, the two types of data from filtered or unfiltered trajectories converge in these long time regimes. Both, the excess-proton identity auto correlations in Supplementary Fig. 15a and the hydronium identities in Supplementary Fig. 15b show a clear concentration dependence, with longer decay times for higher concentrations. On short time scales the excess-proton identity auto correlations largely decay within 0.1 ps to a value of 0.5, whereas

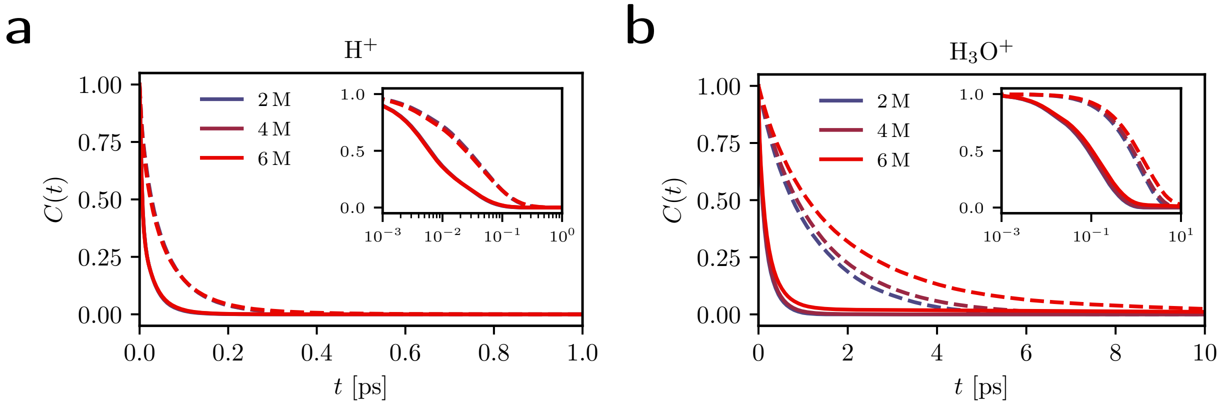

Supplementary Figure 16. Continuous identity auto-correlation functions eq. (24) of the excess protons (a) and hydronium oxygens (b) obtained from ab initio MD simulations of HCl solutions at various concentrations. The correlations functions are computed from filtered (solid colored lines) and unfiltered trajectories (broken colored lines), see text for details. The data is shown on a logarithmic time axis in the insets.

the hydronium identity auto correlations decay to a value of 0.5 only after about 1 ps to 2 ps. Furthermore, a slight peak in the hydronium identity auto-correlation of the unfiltered trajectories (solid colored lines in Supplementary Fig. 15b) at about 0.025 ps indicates back-and-forth transfer of the excess proton in the transient $H_5O_2^+$ cluster occurring at about twice the transfer-path time, $\tau_{\mathrm{TP}} = 12.6$ fs to 14.3 fs reported in the main text. Similarly, a peak in the excess-proton identity auto correlations of the unfiltered trajectories (solid colored lines in Supplementary Fig. 15a) at about 0.010 ps indicates the time scale of excess-proton rattling within a single hydronium ion, referred to as 'special pair dance' in the literature [8, 9].

Next, to focus on the fast time scales of the correlations, the continuous identity auto-correlation functions according to eq. (24) of the excess protons and of the hydronium oxygens are given in Supplementary Figs. 16a and b with the same data shown on logarithmic time axes in the insets. Again, the data is shown in each plot as computed from filtered (broken colored lines) and unfiltered trajectories (solid colored lines). Note, that for the computation of these continuous identity auto correlations, configurations where the excess-proton is located between the oxygen atom and a chloride ion are excluded. These configurations obviously produce spurious long-time auto correlations but have been analyzed to make up only 5% of the total trajectory lengths of all excess protons even at the highest concentration of 6 M,

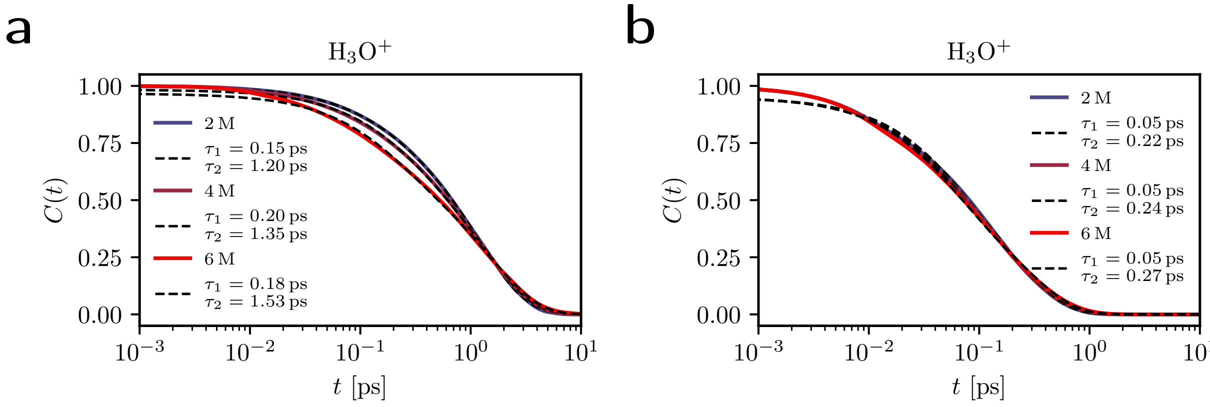

Supplementary Figure 17. Continuous identity auto-correlation functions eq. (24) of the hydronium oxygens obtained from ab initio MD simulations of HCl solutions at various concentrations (already shown in Supplementary Fig. 16b). The correlation functions are computed from filtered (a) and unfiltered trajectories (b), see text for details. Each curve is fitted to a sum of two decaying exponentials with the time scales reported in the legends and shown as black broken lines.

which is discussed in detail in Supplementary Note 4. The excess-proton continuous identity auto correlations decay fully within 0.3 ps and the hydronium-oxygen identity continuous auto-correlations within 10 ps. In contrast to the data presented in Supplementary Fig. 15a and b, while the hydronium identity auto correlations in Supplementary Fig. 16b show again a clear concentration dependence, with longer decay times for higher concentrations, such a dependence is not visible for the excess-proton identity auto correlations in Supplementary Fig. 16a.

The time scales of the hydronium continuous identity auto correlations have been interpreted to be consistent with the time scales of the anisotropy decay observed in 2D IR experiments [19, 38]. Fits to these auto correlations with the sum of two decaying exponentials are therefore given as broken black lines, together with the original data from Supplementary Fig. 16b repeated as solid colored lines in Supplementary Fig. 17a (for the filtered trajectories) and b (for the unfiltered trajectories). The long time-scales of the bi-exponential fits to the correlations of the filtered trajectories, shown in 17a, reach from 1.2 ps to 1.53 ps, increasing with concentration. These time scales match the time scales reported for uni-directional proton transfer rather well (see Supplementary Tab. IV). With regard to the results reported in Supplementary Note 8, where the long-time diffusion constants are found to be too small by a factor of about four when compared to experiment, one would expect the time scales

for uni-directional proton transfer to be longer by the same factor. However, the present analysis excludes configurations involving chloride ions, which presumably are characterized by longer decay times. The diffusion constants on the other hand would have to be split in vehicular diffusion, due to translation of hydronium ions, and the jump diffusion, due to uni-directional proton transfer, to allow for a better comparison to the time scales of the auto correlations.

When regarding the long time-scales of the bi-exponential fits to the correlations of the unfiltered trajectories, shown in Supplementary Fig. 17b, which reach from 0.22 ps to 0.27 ps, increasing with concentration, we find them to match perfectly the mean proton-transfer waiting times reported in the main text and in Supplementary Fig. 14. The continuous hydronium identity auto correlation therefore presents an alternative and intuitive interpretation for the proton-transfer waiting times that we discuss in the main text. In case that back-and-forth fluctuations of the excess-protons between two oxygen atoms within 0.5 ps are filtered from the trajectories, the continuous hydronium identity auto correlation in Supplementary Fig. 17a decays on time scales that match experimental spectroscopic anisotropy decays and have been interpreted as uni-directional proton transfer.

Supplementary Note 8:

Large-scale excess-proton diffusion

For the analysis of diffusion of the excess protons on long time scales, the principle excess-proton identification and selection scheme detailed in Supplementary Methods 2 needs to be augmented to identify joint excess-proton trajectories throughout the whole simulation trajectory. For this, excess protons are first identified based on a geometric criterion: after assembling at each time step the water molecules for each oxygen atom with the closest two hydrogen atoms, the remaining least associated protons form hydronium ions with their respective closest water molecules. We thereby obtain at each time step a total number of excess protons N_{H^+} equivalent to the number of chloride ions in the simulations. The trajectories of the excess protons are then stitched together to a total of N_{H^+} trajectories, each of the length of the whole simulation. In contrast to Supplementary Methods 2, protons that reside between an oxygen and a chloride atom are included in this analysis. The procedure obviously introduces jumps in the joint trajectories whenever an excess proton changes identity, which is a manifestation of the Grotthuss' process. However, rapid spurious jumps within the same hydronium ion, the 'special pair dance' [8, 9], are filtered from the trajectories by the following procedure: within each hydronium ion, the candidate proton that either performs the next transfer to another water molecule or is the next to be identified as an excess proton while neighboring a chloride atom, remains the excess proton (a procedure

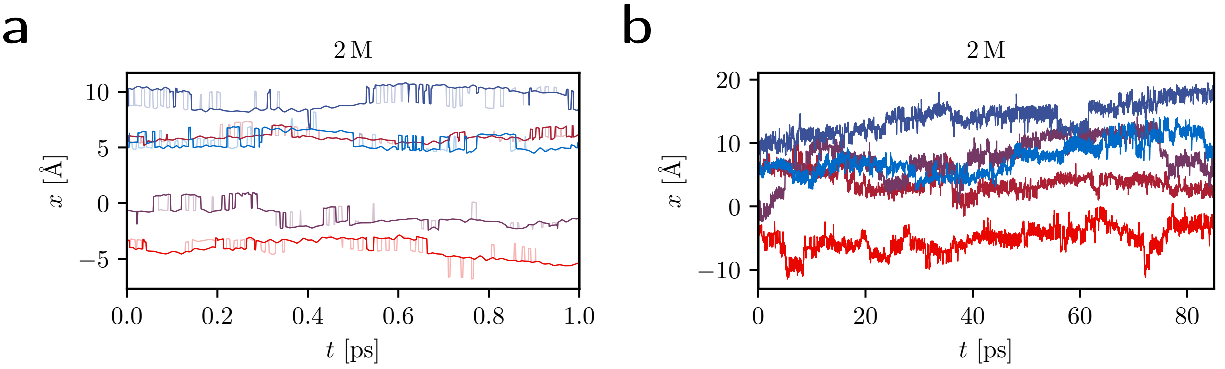

Supplementary Figure 18. Examples of five joint trajectories of excess protons in 2 M HCl solution on short (a) and long time scales (b). In a, the pale colored lines indicate the spurious jumps, resulting from the 'special pair dance', that are removed from the trajectories (see text for details).

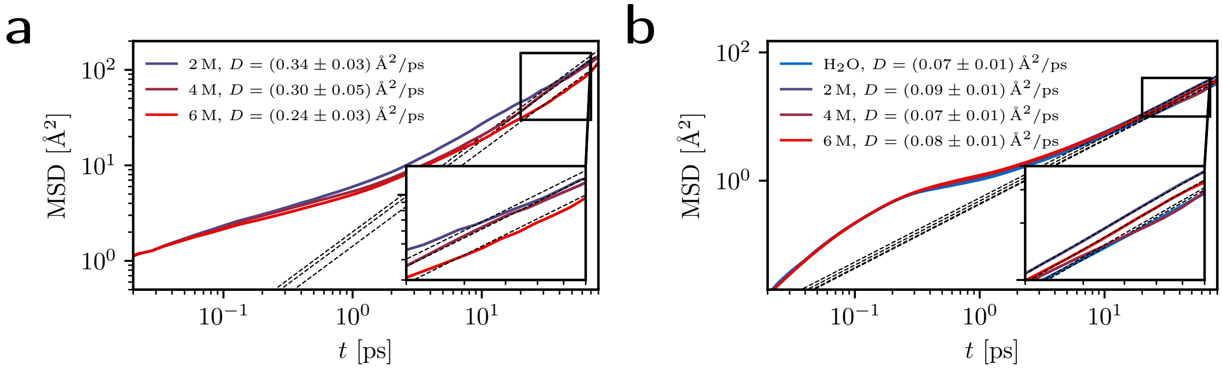

Supplementary Figure 19. MSDs (mean squared displacements) in the lab frame, $\langle|\mathbf{r}(t) - \mathbf{r}(0)|^2\rangle$, of the excess protons (a) and the oxygen atoms (b) computed from the simulation trajectories of HCl solutions at three concentrations. In b, the MSD of the oxygen atoms in the pure-water simulations is shown as well. Errors of the mean are indicated by the line widths and taken from standard deviations computed over the individual excess-proton and oxygen-atom joint trajectories. Linear fits are shown as black broken lines, which are fitted in the long time regimes, 20 ps to 80 ps, which is the range that is also shown enlarged in the insets.

that was also used by Calio et al. [9]). Lastly, the trajectories are unwrapped over the periodic boundary conditions. Some trajectories of excess protons produced by this protocol are illustrated in Supplementary Fig. 18a and b along a single Cartesian coordinate and on two different time scales. Additionally in Supplementary Fig. 18a, the pale colored lines show the trajectories before removal of the 'special pair dance'.

Subsequently, the joint trajectories are used to calculate mean squared displacements (MSDs), $\langle|\mathbf{r}(t) - \mathbf{r}(0)|^2\rangle$, of the excess protons in the lab frame. The MSDs averaged over all excess protons are shown in Supplementary Fig. 19a for simulations of HCl solutions at three different concentrations and compared to the MSDs computed for the oxygen atoms representative of the water molecules in Supplementary Fig. 19b. In general, the MSD is expected to show inertial scaling, MSD $\sim$ t^2, for short time scales and diffusive scaling, MSD $\sim$ t, for long time scales. Both regimes are well visible in Supplementary Fig. 19b for the oxygen atoms. For the excess protons the inertial regime is perturbed by the jumps in the joint trajectories, caused by changes of the excess-proton identities.

The diffusion constant D, which is an experimentally observed quantity, is related to the

MSD by

$$D = \frac{1}{6t}\langle|\mathbf{r}(t) - \mathbf{r}(0)|^2\rangle, \tag{25}$$

and computed by a least-squares fit of the slopes in the diffusive regime between 20 ps to 80 ps. The diffusion constants are reported in the legends of Supplementary Figs. 19a and b with statistical errors from the linear fits.

To access the accuracy of these diffusion constants, we first focus on the values for the oxygen atoms. Within the error the same diffusion constant of about $D_{\mathrm{O}} = 0.08\,\text{Å}^2/\text{ps}$ was obtained in all four simulations. While this value is significantly smaller than the experimental value of $D_{\mathrm{O}} = 0.23\,\text{Å}^2/\text{ps}$ [48, 49], it is well known that specifically the BLYP exchange-correlation functional in our simulation approach produces too small diffusion constants. In agreement with our results, various studies of water utilizing ab initio MD together with the BLYP exchange-correlation functional, reported diffusion constants in the range of $0.04\,\text{Å}^2/\text{ps}$ to $0.11\,\text{Å}^2/\text{ps}$ for comparable setups to ours, as reviewed recently [50].

When regarding the excess proton diffusion constant, it has to be noted that it is known to be heavily concentration and temperature dependent in experiments [23]. In the dilute limit, the experimental diffusion constant of the excess proton, $D_{\mathrm{H+}} = 0.94\,\text{Å}^2/\text{ps}$, is much higher than the experimental diffusion constant of water by a factor of $D_{\mathrm{H+}}/D_{\mathrm{O}} = 4.1$, an observation that corroborates Grotthuss' hypothesis [51]. However, this factor drops to about 3.0 at 2 M and 1.5 at 6 M [23]. This trend was qualitatively captured in self-consistent iterative multistate empirical valence bond (SCI-MS-EVB) simulations of HCl [16, 17]. Both studies obtained values between $D_{\mathrm{H+}} = 0.2\,\text{Å}^2/\text{ps}$ to $0.3\,\text{Å}^2/\text{ps}$ around 1 M and $D_{\mathrm{H+}} = 0.15\,\text{Å}^2/\text{ps}$ to $0.20\,\text{Å}^2/\text{ps}$ around 3 M, compared to a value of $D_{\mathrm{H+}} = 0.37\,\text{Å}^2/\text{ps}$ in the dilute limit (one excess proton in 256 waters) [13]. While a similar trend is also indicated by our simulation data, the errors are too large to draw definite conclusions. The obtained diffusion constants for the excess protons, $D_{\mathrm{H+}} = 0.24\,\text{Å}^2/\text{ps}$ to $0.34\,\text{Å}^2/\text{ps}$, are smaller than the experimental value. However, the ratios $D_{\mathrm{H+}}/D_{\mathrm{O}} = 0.34/0.09 = 3.8 \pm 0.8$ for 2 M, $D_{\mathrm{H+}}/D_{\mathrm{O}} = 0.30/0.07 = 4.3 \pm 1.3$ for 4 M and $D_{\mathrm{H+}}/D_{\mathrm{O}} = 0.24/0.08 = 3.0 \pm 0.8$ for 6 M, that are observed in our simulations, seem in satisfactory agreement with experiments. Similar ab initio simulation setups to ours but using a single excess proton in a box of water molecules obtained diffusion constants of $D_{\mathrm{H+}} = 0.3\,\text{Å}^2/\text{ps}$ to $0.6\,\text{Å}^2/\text{ps}$ [22] and of $D_{\mathrm{H+}} = 0.3\,\text{Å}^2/\text{ps}$ to $0.8\,\text{Å}^2/\text{ps}$ [19]. A study employing Car-Parrinello molecular-dynamics (CPMD) simulations

of 1.7 M HCl solutions and the PBE exchange-correlation functional found $D_{\mathrm{H^+}} = 0.9\,\text{Å}^2/\mathrm{ps}$ to $1.1\,\text{Å}^2/\mathrm{ps}$ compared to $D_{\mathrm{O}} = 0.04\,\text{Å}^2/\mathrm{ps}$ to $0.07\,\text{Å}^2/\mathrm{ps}$ for the water molecules [18]. An older study of a single excess proton in a box of 64 water molecules using CPMD with the BLYP functional obtained $D_{\mathrm{H^+}} = 0.05\,\text{Å}^2/\mathrm{ps}$ to $0.08\,\text{Å}^2/\mathrm{ps}$ compared to $D_{\mathrm{O}} = 0.02\,\text{Å}^2/\mathrm{ps}$ to $0.06\,\text{Å}^2/\mathrm{ps}$ for the water molecules [52]. The inclusion of nuclear quantum effects in simulations has been shown to significantly increase the obtained excess-proton diffusion constants [13].

We conclude that the accurate estimation of diffusion constants for the excess proton remains challenging, which is seen from the wide spread of results obtained in previous studies. Our results for the diffusion constants appear reasonable when compared to previously reported values and specifically the ratios $D_{\mathrm{H^+}}/D_{\mathrm{O}} = 3.0$ to 4.3 are in good agreement with experiment.

Supplementary Note 9: Hydrogen-bond structure

The hydrogen bond (HB) structure of the water molecules in the first hydration shell of the excess proton has been shown to play an important role for when and where the excess proton moves [11, 13, 18, 22]. In this Supplementary Note the HB structure around the excess protons in HCl solutions obtained in our ab initio simulations is discussed using some previously established methods. We follow the standard criterion and define HBs to be present when the distance between two oxygens, i.e. the donor and the acceptor of the HB, is $< 3.5\,\text{Å}$, and the angle between the vector connecting the two oxygens and the vector connecting the donor oxygen with the hydrogen atom is $< 30°$ [47].

Hydrogen-bond asymmetry

Napoli et al. [11] introduced the HB asymmetry ϕ as a measure to identify the excess proton among the candidate protons in a hydronium ion. First, each water molecule is assigned a coordination number as the difference of the number of acceptor HBs minus the number of donor HBs. Then ϕ is defined for each candidate proton at each time step as the coordination number of the closest neighboring water molecule minus the average of the coordination numbers of the closest water molecules of the other candidate protons of the same hydronium ion. By construction, the sum of ϕ within a hydronium ion is zero. Napoli et al. [11] found that protons with strongly negative values of ϕ show the typical spectral signatures associated with excess protons. We applied this measure to our simulation data, specifically to the joint trajectories of the excess protons that were prepared for the analysis of the long time diffusion in Supplementary Note 8 and from which the spurious 'special pair dance' was removed.

Normalized distributions of ϕ over the whole simulations are shown in Supplementary Fig. 20a with mean values given in the legend. The mean values are clearly negative and we therefore conclude that this criterion agrees with our excess-proton identification scheme. To study the relation of HB structure with proton transfer, we filter the trajectories in time and regard the normalized distributions of ϕ values around the transfer events, when an excess proton changes its closest oxygen. Distributions of ϕ during 20 fs (40 time steps) before and after

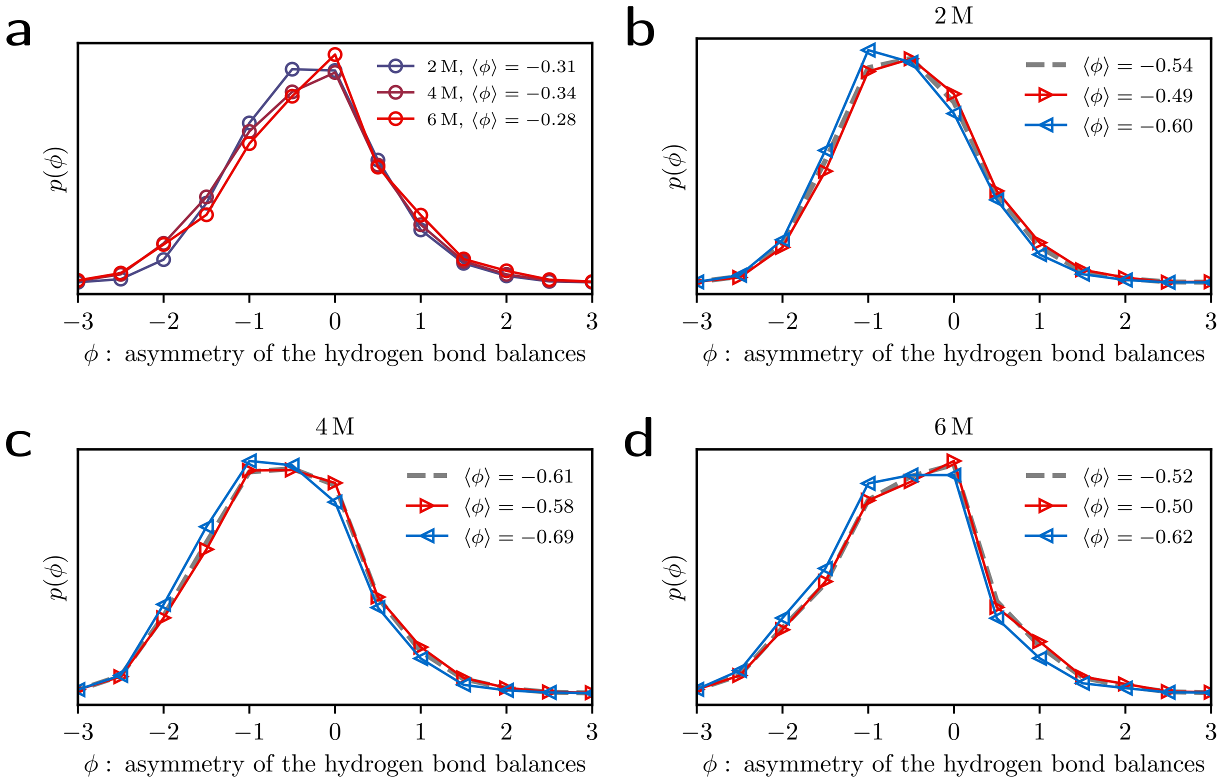

Supplementary Figure 20. Normalized distributions of the hydrogen bond asymmetries, denoted as ϕ, of the excess proton trajectories from ab initio MD simulations of HCl solutions at various concentrations. See text for definition and details. Mean values of the distributions are reported in the legends. **a:** Distributions over the whole trajectories. **b–d:** Distributions of ϕ during 20 fs before and after any transfer event (grey broken lines), i.e. when the excess proton changes the closest oxygen, and distributions of ϕ around each uni-directional transfer event, which are split into the 20 fs before, corresponding to the donor oxygen of the proton transfer event (blue and left pointing triangles) and the 20 fs after, corresponding to the acceptor oxygen (red and right pointing triangles).

a transfer event are given in Supplementary Fig. 20b–d as grey broken lines with mean values reported in the legends. To further elucidate the data, we applied the same filter before and after each uni-directional transfer event. Uni-directional here refers to transfer events, that are not followed by a fast return back to the original oxygen atom within the following 50 fs (100 time steps). By only regarding these uni-directional events, we can discriminate different HB configurations around the donor oxygen of the proton-transfer

event, i.e. before the uni-directional transfer (shown in blue with left pointing triangles), and the acceptor oxygen, i.e. after the uni-directional transfer (shown in red with right pointing triangles), which show distinct distributions in Supplementary Fig. 20b–d. The mean values of the distributions before a transfer are more negative than the corresponding averages in Supplementary Fig. 20a, indicating that on average a more negative value of ϕ precedes an imminent transfer event. Therefore, ϕ actually seems to be a useful predictor for proton transfer.

The fourth hydrogen bond

Inspired by Tse et al. [22], Biswas et al. [13] and Fischer and Gunlycke [18] investigated the role of a fourth water molecule hydrogen-bonded to the hydronium ion. Presumably, its presence determines whether an excess proton would transfer to a different oxygen atom [13] and in particular whether it is likely to return to the original oxygen atom, i.e. to perform back-and-forth transfers, or not [18]. In their simulations of 1.7 M HCl solutions, Fischer and Gunlycke [18] observed a higher return probability (67 %) if the hydronium ion was

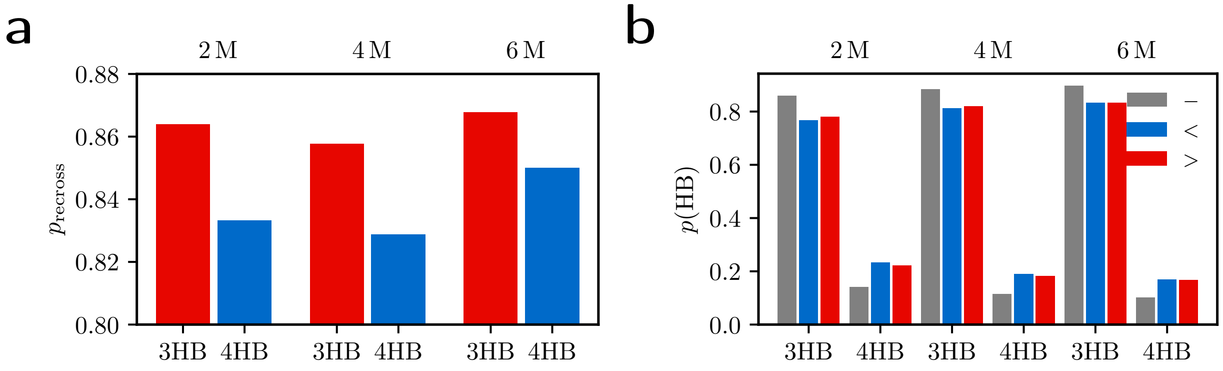

Supplementary Figure 21. Correlations between the existence of a fourth hydrogen bond of a hydronium ion (4HB) and the return probability of the excess proton, as observed in ab initio MD simulations of HCl solutions at various concentrations. **a:** Probability of the excess proton to return across the mid plane between the oxygens within the following 50 fs. **b:** Time-averaged probabilities of all hydronium ions for the fourth hydrogen bond to exist (4HB) or not (3HB), as obtained from the whole simulation (grey, no hatching) and during the 20 fs before (blue) and after (red) each uni-directional transfer event.

coordinated with only three donor HBs as compared to four HBs (53 %), the additional one being an accepted HB from a fourth water molecule. In accordance with previous works, the fourth HB is defined to be present if the vector connecting the hydronium oxygen and the hydrogen atom of the fourth water molecule has a length of less than 2.6 Å and points at an angle of less than 35° with respect to the normal of the plane spanned by the three hydrogen nuclei of the hydronium ion [18, 22]. The probability of a return within the following 50 fs (100 time steps) upon any transfer across the mid plane between the oxygens obtained in our simulations is illustrated in Supplementary Fig. 21a depending on whether the donor hydrogen ion is accepting a HB from a fourth water molecule (4HB, shown in blue with hatching) or not (3HB, shown in red without hatching). The data shows the clear trend that the return probability is reduced if a fourth HB is present, in agreement with previous studies [18]. However, even though this correlation is discernible in our data, the mechanism does not appear to be a dominant driver for proton transfer in HCl solutions. This follows from Supplementary Fig. 21b, where the probability of observing the fourth HB is plotted for three different time averages; the averages over the whole simulations of all hydronium ions are given in grey, the averages over the 20 fs (40 time steps) before and after each uni-directional transfer event, i.e. without return, are given in blue and red respectively. Throughout the data, the time-averaged probability of a fourth HB is only about 10–20%. Before and after uni-directional transfer events this probability is significantly increased but it is still smaller than the probability of a uni-directional transfer event happening without the presence of a fourth HB.

We confirm that HB structure is highly correlated with the excess-proton transfer dynamics and the presented comparison with previous studies strengthens the existing hypotheses, that the HB asymmetry ϕ or the fourth water molecule are useful descriptors.

Supplementary References

[1] Bertie, J. E. & Lan, Z. Infrared intensities of liquids XX: The intensity of the OH stretching band of liquid water revisited, and the best current values of the optical constants of H_2O(l) at 25°C between 15,000 and 1 cm^{-1}. *Appl. Spectrosc.* **50**, 1047 (1996).

[2] Jackson, J. D., *Classical electrodynamics* (John Wiley & Sons, Inc., 1999).

[3] Lucarini, V., Saarinen, J. J., Peiponen, K.-E. & Vartiainen, E. M., *Kramers-Kronig relations in optical materials research*, vol. 110 (Springer Science & Business Media, 2005).

[4] Goplen, T. G., Cameron, D. G. & Jones, R. N. The Control of Errors in Infrared Spectrophotometry. VI. The Evaluation of Optical Constants by Combined Transmission and Attenuated Total Reflection Measurements. *Appl. Spectrosc.* **34**, 652 (1980).

[5] Mayerhöfer, T. G. & Popp, J. Quantitative Evaluation of Infrared Absorbance Spectra – Lorentz Profile versus Lorentz Oscillator. *ChemPhysChem* **20**, 31 (2019).

[6] Schienbein, P., Schwaab, G., Forbert, H., Havenith, M. & Marx, D. Correlations in the solute-solvent dynamics reach beyond the first hydration shell of ions. *J. Phys. Chem. Lett.* **8**, 2373 (2017).

[7] Marx, D., Tuckerman, M. E., Hutter, J. & Parrinello, M. The nature of the hydrated excess proton in water. *Nature* **397**, 601 (1999).

[8] Markovitch, O. *et al.* Special pair dance and partner selection: Elementary steps in proton transport in liquid water. *J. Phys. Chem. B* **112**, 9456 (2008).

[9] Calio, P. B., Li, C. & Voth, G. A. Resolving the Structural Debate for the Hydrated Excess Proton in Water. *J. Am. Chem. Soc.* **143**, 18672 (2021).

[10] Wiener, N. Generalized harmonic analysis. *Acta Math.* **55**, 117 (1930).

[11] Napoli, J. A., Marsalek, O. & Markland, T. E. Decoding the spectroscopic features and time scales of aqueous proton defects. *J. Chem. Phys.* **148**, 222833 (2018).

[12] Markland, T. E. & Ceriotti, M. Nuclear quantum effects enter the mainstream. *Nat. Rev. Chem.* **2**, 0109 (2018).

[13] Biswas, R., Tse, Y. L. S., Tokmakoff, A. & Voth, G. A. Role of Presolvation and Anharmonicity in Aqueous Phase Hydrated Proton Solvation and Transport. *J. Phys. Chem. B* **120**, 1793

(2016).

[14] Baer, M. D., Fulton, J. L., Balasubramanian, M., Schenter, G. K. & Mundy, C. J. Persistent ion pairing in aqueous hydrochloric acid. *J. Phys. Chem. B* **118**, 7211 (2014).

[15] Fulton, J. L. & Balasubramanian, M. Structure of Hydronium (H_3O^+)/ Chloride (Cl^-) Contact Ion Pairs in Aqueous Hydrochloric Acid Solution : A Zundel-like. *J. Am. Chem. Soc.* **132**, 12597 (2010).

[16] Xu, J., Izvekov, S. & Voth, G. A. Structure and dynamics of concentrated hydrochloric acid solutions. *J. Phys. Chem. B* **114**, 9555 (2010).

[17] Calio, P. B., Li, C. & Voth, G. A. Molecular Origins of the Barriers to Proton Transport in Acidic Aqueous Solutions. *J. Phys. Chem. B* **124**, 8868 (2020).

[18] Fischer, S. A. & Gunlycke, D. Analysis of Correlated Dynamics in the Grotthuss Mechanism of Proton Diffusion. *J. Phys. Chem. B* **123**, 5536 (2019).

[19] Arntsen, C., Chen, C., Calio, P. B., Li, C. & Voth, G. A. The hopping mechanism of the hydrated excess proton and its contribution to proton diffusion in water. *J. Chem. Phys.* **154**, 194506 (2021).

[20] Kundu, A. *et al.* Hydrated Excess Protons in Acetonitrile/Water Mixtures: Solvation Species and Ultrafast Proton Motions. *J. Phys. Chem. Lett.* **10**, 2287 (2019).

[21] Carpenter, W. B. *et al.* Decoding the 2D IR spectrum of the aqueous proton with high-level VSCF/VCI calculations. *J. Chem. Phys.* **153**, 124506 (2020).

[22] Tse, Y. L. S., Knight, C. & Voth, G. A. An analysis of hydrated proton diffusion in ab initio molecular dynamics. *J. Chem. Phys.* **142**, 014104 (2015).

[23] Dippel, T. & Kreuer, K. D. Proton transport mechanism in concentrated aqueous solutions and solid hydrates of acids. *Solid State Ionics* **46**, 3 (1991).

[24] Daly, C. A. *et al.* Decomposition of the Experimental Raman and Infrared Spectra of Acidic Water into Proton, Special Pair, and Counterion Contributions. *J. Phys. Chem. Lett.* **8**, 5246 (2017).

[25] Fournier, J. A., Carpenter, W. B., Lewis, N. H. & Tokmakoff, A. Broadband 2D IR spectroscopy reveals dominant asymmetric $H_5O_2{}^+$ proton hydration structures in acid solutions. *Nat. Chem.* **10**, 932 (2018).

[26] Biswas, R., Carpenter, W., Fournier, J. A., Voth, G. A. & Tokmakoff, A. IR spectral assignments for the hydrated excess proton in liquid water. *J. Chem. Phys.* **146**, 154507 (2017).

[27] Thämer, M., De Marco, L., Ramasesha, K., Mandal, A. & Tokmakoff, A. Ultrafast 2D IR spectroscopy of the excess proton in liquid water. *Science* **350**, 78 (2015).

[28] Hansen, J.-P. & McDonald, I. R., *Theory of simple liquids: with applications to soft matter* (Academic press, 2013).

[29] Carpenter, W. B., Lewis, N. H., Fournier, J. A. & Tokmakoff, A. Entropic barriers in the kinetics of aqueous proton transfer. *J. Chem. Phys.* **151**, 034501 (2019).

[30] VandeVondele, J. & Hutter, J. Gaussian basis sets for accurate calculations on molecular systems in gas and condensed phases. *J. Chem. Phys.* **127**, 114105 (2007).

[31] VandeVondele, J. *et al.* Quickstep: Fast and accurate density functional calculations using a mixed Gaussian and plane waves approach. *Comput. Phys. Commun.* **167**, 103 (2005).

[32] Brünig, F. N., Hillmann, P., Kim, W. K., Daldrop, J. O. & Netz, R. R., Proton-transfer spectroscopy beyond the normal-mode scenario (2021), Preprint at arXiv:2109.08514.

[33] Sauer, J. & Döbler, J. Gas-phase infrared spectrum of the protonated water dimer: Molecular dynamics simulation and accuracy of the potential energy surface. *ChemPhysChem* **6**, 1706 (2005).

[34] Agmon, N. The Grotthuss mechanism. *Chem. Phys. Lett.* **244**, 456 (1995).

[35] Meiboom, S. Nuclear magnetic resonance study of the proton transfer in water. *J. Chem. Phys.* **34**, 375 (1961).

[36] Woutersen, S. & Bakker, H. J. Ultrafast vibrational and structural dynamics of the proton in liquid water. *Phys. Rev. Lett.* **96**, 138305 (2006).

[37] Dahms, F., Fingerhut, B. P., Nibbering, E. T., Pines, E. & Elsaesser, T. Large-amplitude transfer motion of hydrated excess protons mapped by ultrafast 2D IR spectroscopy. *Science* **357**, 491 (2017).

[38] Carpenter, W. B., Fournier, J. A., Lewis, N. H. & Tokmakoff, A. Picosecond Proton Transfer Kinetics in Water Revealed with Ultrafast IR Spectroscopy. *J. Phys. Chem. B* **122**, 2792 (2018).

[39] Yuan, R. *et al.* Tracking Aqueous Proton Transfer by Two-Dimensional Infrared Spectroscopy and ab Initio Molecular Dynamics Simulations. *ACS Cent. Sci.* **5**, 1269 (2019).

[40] Fischer, S. A., Dunlap, B. I. & Gunlycke, D. Correlated dynamics in aqueous proton diffusion. *Chem. Sci.* **9**, 7126 (2018).

[41] Roy, S. *et al.* Resolving Heterogeneous Dynamics of Excess Protons in Aqueous Solution with

Rate Theory. *J. Phys. Chem. B* **124**, 5665 (2020).

[42] Hänggi, P., Talkner, P. & Borkovec, M. Reaction-rate theory: Fifty years after Kramers. *Rev. Mod. Phys.* **62**, 251 (1990).

[43] Ciccotti, G., Ferrario, M., Hynes, J. T. & Kapral, R. Dynamics of ion pair interconversion in a polar solvent. *J. Chem. Phys.* **93**, 7137 (1990).

[44] Roy, S., Baer, M. D., Mundy, C. J. & Schenter, G. K. Reaction Rate Theory in Coordination Number Space: An Application to Ion Solvation. *J. Phys. Chem. C* **120**, 7597 (2016).

[45] Kappler, J., Daldrop, J. O., Brünig, F. N., Boehle, M. D. & Netz, R. R. Memory-induced acceleration and slowdown of barrier crossing. *J. Chem. Phys.* **148**, 014903 (2018).

[46] Hassanali, A., Giberti, F., Cuny, J., Kühne, T. D. & Parrinello, M. Proton transfer through the water gossamer. *Proc. Natl. Acad. Sci. U. S. A.* **110**, 13723 (2013).

[47] Luzar, A. & Chandler, D. Hydrogen-bond kinetics in liquid water. *Nature* **379**, 55 (1996).

[48] Mills, R. Self-diffusion in normal and heavy water in the range 1-45°. *J. Phys. Chem.* **77**, 685 (1973).

[49] Harris, K. R. & Woolf, L. A. Pressure and temperature dependence of the self diffusion coefficient of water and oxygen-18 water. *J. Chem. Soc. Faraday Trans. 1* **76**, 377 (1980).

[50] Gillan, M. J., Alfè, D. & Michaelides, A. Perspective: How good is DFT for water? *J. Chem. Phys.* **144**, 130901 (2016).

[51] Roberts, N. & Northey, H. L. Hydrogen Ion Mobility in Aqueous Electrolyte Solutions. *J. Chem. Soc. Faraday Trans. 1* **68**, 1528 (1972).

[52] Izvekov, S. & Voth, G. A. Ab initio molecular-dynamics simulation of aqueous proton solvation and transport revisited. *J. Chem. Phys.* **123**, 044505 (2005).
